# Soil phosphorus availability drives decade-scale stability across life stages in a subtropical forest

**DOI:** 10.1126/sciadv.aec5351

**Published:** 2026-04-24

**Authors:** Ganxin Feng, Dong Dai, Yi Zheng, David Johnson, David F. R. P. Burslem, Shixiao Yu, Xubing Liu, Minxia Liang

**Affiliations:** ^1^ State Key Laboratory of Biocontrol, School of Ecology, Sun Yat-sen University, Shenzhen Campus, Shenzhen 518107, China.; ^2^ South China Botanical Garden, Chinese Academy of Science, Guangzhou 510650, China.; ^3^ Lancaster Environment Centre, Lancaster University, Lancaster LA1 4YQ, UK.; ^4^ Interdisciplinary Institute and School of Biological Sciences, University of Aberdeen, Aberdeen AB24 3UU, UK.; ^5^ State Key Laboratory of Biocontrol, School of Life Science, Sun Yat-sen University, Guangzhou 510275, China.

## Abstract

The potential of tree diversity to stabilize community productivity is well established. However, how different forms of soil nutrients regulate tree diversity and stability across life stages remains unclear. Using high-resolution soil nutrient mapping and inventory data from a subtropical forest in southern China, we examined how different forms of soil nitrogen (N) and phosphorus (P) regulate tree diversity and stability across seedling and adult stages. Tree diversity was consistently associated with greater stability, with both seedling richness and adult evenness driving stability, respectively. Inorganic P alleviated interspecific competition, and enhanced seedling richness and stability, but suppressed adult asynchrony and stability. Conversely, organic P was consistently associated with reduced diversity and stability across both stages, coupled with strengthened interspecific competition. Inorganic and organic N exhibited comparatively weaker influences on diversity and stability. These findings highlight the importance of considering tree life stage and soil P form in managing and conserving forest ecosystems.

## Teaser

Long-term tree demography and high-resolution soil mapping reveal soil P availability shapes forest stability across life stages.

## INTRODUCTION

Ecosystem stability is a cornerstone of ecological functioning and the continued provision of various ecosystem services (*1*). However, the increasing pressure from anthropogenic activities and environmental changes threatens the temporal stability of ecosystems, including diversity, ecosystem functioning, and ecosystem services (*2*, *3*). Understanding the drivers of temporal stability is crucial for developing effective conservation and management strategies (*4*, *5*). Soil nutrients play a pivotal role in shaping ecosystem stability because they are fundamental regulators of plant growth, community composition, and ecosystem functions. The availability of essential nutrients such as nitrogen (N) and phosphorus (P) can directly affect the productivity and diversity of plant communities (*6*, *7*), which in turn affects the temporal stability of ecosystem functioning over time (*3*, *8*–*12*). However, existing studies have primarily focused on controlled experiments in managed grassland systems dominated by herbaceous plants (*8*, *10*–*15*). While these studies have provided valuable insights, their findings may not apply directly to forest ecosystems, where the dynamics and interactions are more complex because of the tree community’s high diversity and longevity.

Moreover, most forest stability studies focus solely on specific life stages, such as seedlings (*9*) or adult trees (*16*–*18*), overlooking the differences in stability drivers across these stages. Yet, in reality, tree communities represent a continuum of tree ages and life stages, and trees at different stages of their life cycle play different ecological roles. For instance, seedlings drive community recruitment and regeneration (*19*), while adults contribute substantially to carbon sequestration (*20*) and overall forest structure (*21*). Differences in nutrient acquisition strategies and metabolic demands suggest that different life stages of trees may differ in their responses to changes in soil nutrient availabilities (*22*–*24*). Despite their ecological significance, the effects of soil nutrient availability on the temporal stability of forest communities across life stages remain largely unexplored. Addressing this knowledge gap is crucial for advancing our understanding of forest ecosystem functioning and dynamics in the face of global change.

Ecosystem temporal stability refers to the temporal invariability of specific ecosystem functions based on one or more property (such as productivity), calculated as its temporal mean divided by its standard deviation (SD) over time (*25*). A recent theoretical framework proposes a comprehensive understanding of ecosystem (or community) temporal stability by partitioning it into two distinct components: population stability and species asynchrony (*26*, *27*). Greater community stability can emerge from either more stable population dynamics (population stability) or increased species asynchrony, which captures the “temporal incoherence” (*28*) in population dynamics among different species within the community (*5*).

Many studies have shown that greater species richness promotes community stability by enhancing population stability (increased likelihood of the presence of species with higher stability) and species asynchrony (including species with differential responses to environmental changes) (*29*, *30*). However, species evenness, which measures the equitability of species abundance within a community (*31*), is also crucial for stabilizing plant communities, yet it has often been overlooked (*27*, *32*). Previous theories have shown that statistical-averaging effects are dampened by lower evenness (*33*). Lower evenness reduces the stabilizing effects of species richness due to less statistical averaging, as dominant species overshadow the contributions of rare species according to the mass ratio hypothesis (*32*). A higher relative abundance of rare species (e.g., increasing evenness) is expected to increase community temporal stability. Species richness alone may not fully capture the role of species diversity in stabilizing community productivity (*34*, *35*). Moreover, a recent global-scale study revealed that increasing species richness in natural forest communities generally leads to declining species evenness (*36*). Therefore, it is essential to consider both richness and evenness to understand the mechanistic basis of diversity-stability relationships in natural forests.

Soil nutrient availability may influence community temporal stability through their effects on species diversity. Higher nutrient concentrations, such as N enrichment, are often thought to reduce plant diversity through intensified competition for light, soil acidification, and the leaching of essential base cations (*37*, *38*). The N-induced species diversity loss may weaken ecosystem stability by reducing population stability and species asynchrony (*14*). However, most tests of this hypothesis have focused on grassland communities, and less is known about the effects of nutrient availability in forest communities, possibly because the long life span of trees requires longer demographic time series to characterize stability metrics. Moreover, evidence from long-term, factorial forest fertilization experiments suggests that the initial responses triggered by nutrient addition are reflected in adjustment to plant physiology and carbon allocation (e.g., reduced investment in fine roots). Stand-level productivity responses, however, are often nutrient specific and time lagged; these dynamics may differ between deeply shaded seedlings and canopy trees, further motivating life-stage-explicit, long-term assessments of stability in forests (*39*). A long-term N addition experiment in a tropical forest revealed that high soil N concentrations significantly decreased species diversity, particularly among tree seedlings (*37*). Yet, it remains unclear whether loss of tree diversity due to N enrichment affects the stability of tree communities. Moreover, previous studies in subtropical forests have found that N addition has either a negative or a neutral effect on the growth of small trees while promoting the growth of large trees—a pattern likely shaped by N saturation in these forests and the increasing nutrient demands as trees age (*23*). Trees at different life stages may respond differently to nutrient addition due to variations in nutrient foraging capacity. However, the influence of nutrient availability on tree growth dynamics across different life stages remains largely unexplored.

Subtropical forests are typically characterized by P limitation (*40*), primarily due to prolonged weathering and leaching processes that have caused extensive P depletion from the soil profile (*41*, *42*). Approximately 30 to 80% of total P in forest soils exists in organic forms (*43*). Resource partitioning of different forms of soil P among plant species is a key mechanism explaining species coexistence in P-limited regions (*44*, *45*). While previous studies in subtropical forests have shown that the abundance of different P forms is generally associated with plant abundance and growth (*44*, *46*, *47*), the effects of increased P availability or unavailability on tree diversity and productivity stability remain poorly understood. Seedling and sapling populations are particularly sensitive to soil P availability (*48*, *49*), not only because they allocate proportionally more P to growth-related traits (such as specific leaf area, representing the ability to intercept light) under light limitation (*24*, *50*) but also because low light constrains carbon gain and thereby limits root function and nutrient foraging capacity, making shaded juveniles especially prone to nutrient limitation (*39*). Environments rich in inorganic P support more surviving seedlings, enhancing community diversity and stabilizing plant communities over time (*9*). In contrast, canopy trees experience higher light availability that can sustain root function and nutrient acquisition, and long-lived trees tend to allocate nutrients to defense-related traits (such as tissue lignification, leaf toughness, and phenolic concentrations) before growth or productivity responses become detectable, potentially generating delayed or weaker responses at later life stages (*23*, *39*, *50*). Thus, tree diversity and stability across life stages may exhibit different responses to soil P availability.

Community-level diversity and stability patterns could emerge from local interactions among neighboring individuals (*28*). From a coexistence and species-interaction perspective, diversity is maintained when conspecific negative density dependence (CNDD) exceeds heterospecific effects (*51*), whereas shifts toward stronger heterospecific competition can promote dominance and reduce richness. Soil nutrient availability can modify these neighborhood effects by changing resource limitation, nutrient-acquisition strategies, and rhizosphere microbial processes (*52*). Therefore, in addition to evaluating the relationships among soil nutrients, diversity components, and community stability, we test whether different forms of N and P modify conspecific and heterospecific neighborhood effects across life stages. This interaction-based test provides a mechanistic bridge linking nutrient forms to diversity components and community stability.

Here, we use a long-term monitoring dataset of seedling and adult dynamics in a subtropical forest to investigate how soil nutrient forms influence the stability of tree communities across life stages. Specifically, we mainly focus on two questions: (i) Can species diversity (richness and evenness) enhance community temporal stability through population stability or species asynchrony across all life stages? (ii) How do different chemical forms of soil nutrients regulate tree diversity and, in turn, community stability through modifying conspecific and heterospecific neighborhood effects (i.e., density-dependent competition/facilitation) across life stages? We hypothesize that higher soil inorganic P concentrations promote seedling diversity and stability by reducing competition, while adult trees become less dependent on P because of increased investment in defense traits. In contrast, soil with a higher organic P pool may indicate that a larger share of P is associated with organic matter and would require mineralization before uptake, which may shift competitive advantages toward species with particular nutrient-acquisition strategies, potentially promoting dominance and reducing diversity and stability. Similarly, N—especially organic N—may negatively affect diversity and stability by intensifying interspecific competition. Overall, we propose that soil nutrient forms are key regulators of diversity and temporal stability in forest communities.

## RESULTS

### The diversity-stability relationships across life stages

**Fig. 1.**
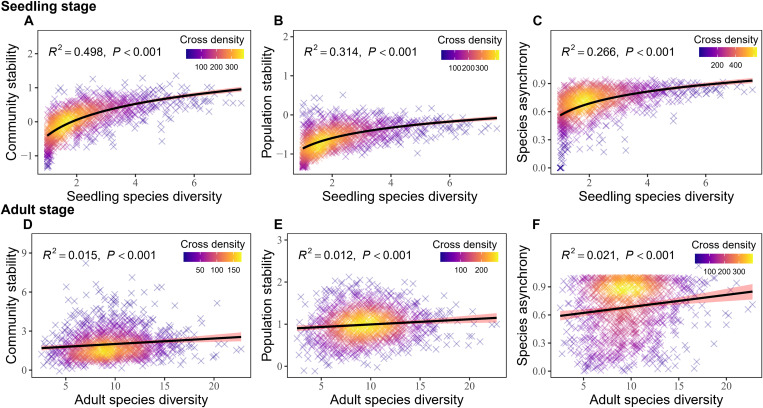
The diversity-stability relationships across life stages. (**A** to **C**) respectively depict community stability, population stability, and species asynchrony in relation to species diversity for the seedling stage, while (**D** to **F**) depict the same relationships for the adult stage. The solid lines represent significant relationships fitted by bivariate linear regression, and the shades are the corresponding 95% confidence intervals. Community stability and population stability were log-transformed using natural logarithm base e. Each cross represents one sampling unit: a 1 m–by–1 m seedling quadrat across the six 1-ha seedling plot (seedling stage) or a 20 m–by–20 m quadrat within the 50-ha plot (adult stage). Crosses are colored by cross density (top-right color legend), with warmer colors indicating higher cross densities. Detailed statistical parameters are provided in table S1.

**Fig. 2.**
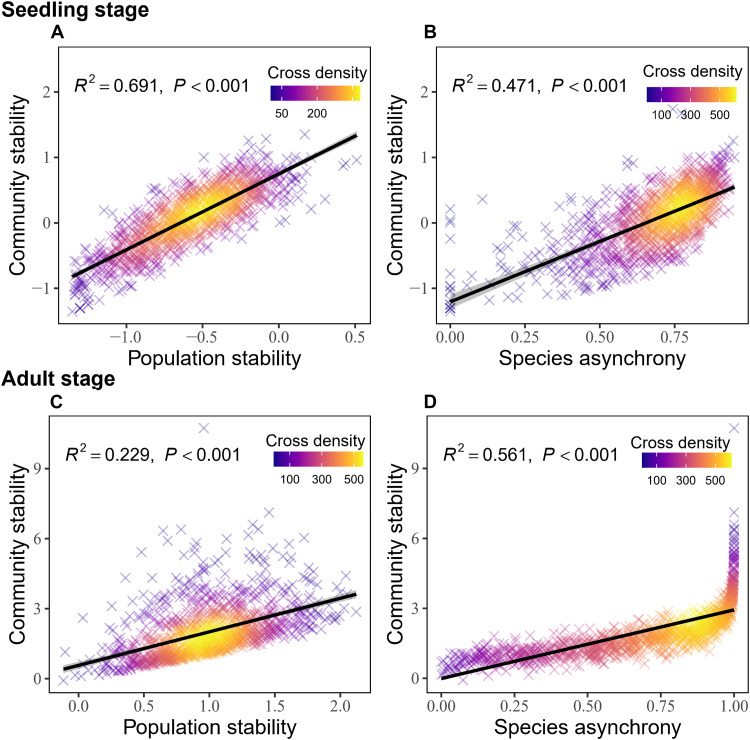
Relationships between population stability, species asynchrony, and community stability across life stages. (**A**) and (**B**) show relationships for the seedling stage: (A) population stability versus community stability; (B) species asynchrony versus community stability. (**C**) and (**D**) show the same relationships for the adult stage: (C) population stability versus community stability; (D) species asynchrony versus community stability. The solid lines represent significant relationships fitted by bivariate linear regression, and the shades are the corresponding 95% confidence intervals. Community stability and population stability were log-transformed using natural logarithm base e. Each cross represents one sampling unit: a 1 m–by–1 m seedling quadrat across the six 1-ha seedling plot (seedling stage) or a 20 m–by–20 m quadrat within the 50-ha plot (adult stage). Crosses are colored by cross density (top-right color legend), with warmer colors indicating higher cross densities. Detailed statistical parameters are provided in table S1.

The diversity-stability relationships varied across life stages. In seedling quadrats, seedling species diversity (measured by the exponential of Shannon’s entropy) showed a strong positive correlation with community stability (temporal stability of community productivity, defined in terms of seedling height growth), population stability, and species asynchrony (Fig. 1, A to C, and table S1). This relationship was primarily driven by seedling species richness. Combined with a higher positive correlation with seedling species diversity [fig. S1A, coefficient of determination (*R*^2^) = 0.915], seedling richness also significantly positively correlated with community stability (table S1; *R*^2^ = 0.464), population stability (table S1; *R*^2^ = 0.324), and species asynchrony (table S1; *R*^2^ = 0.208). In contrast, seedling species evenness showed significant negative relationships with community stability, population stability, and species asynchrony (table S1). These results indicated that the diversity-stability relationship at the seedling stage was driven by seedling richness. In adult quadrats, adult tree species diversity also showed a significant positive relationship with community stability [temporal stability of community productivity, defined in terms of adult aboveground biomass (AGB) growth], population stability, and species asynchrony (Fig. 1, D to F, and table S1), but the relationship between adult tree species diversity and stability was more closely associated with adult tree species evenness. Adult tree species diversity showed a greater correlation with adult tree species evenness (fig. S1D, *R*^2^ = 0.557) compared to adult tree species richness (fig. S1C, *R*^2^ = 0.143). Adult tree species evenness directly affected temporal stability and species asynchrony, while adult tree species richness showed no significant effects (table S1). Thus, at the adult stage, the diversity-stability relationship is driven by evenness rather than richness. In addition, both population stability and species asynchrony explain a substantial fraction of the variation in community stability (Fig. 2, A to D, and table S1). Population stability accounts for more of the variation during the seedling stage (Fig. 2A, *R*^2^ = 0.691), while species asynchrony plays a more significant role in explaining community stability at the adult stage (Fig. 2D, *R*^2^ = 0.561). Overall, a consistently positive diversity-stability relationship was detected across life stages, but the key diversity components regulating stability differed among life stages.

Community composition showed a significant association with the stability gradient at both life stages, although effect sizes differed (table S2). At the seedling stage, composition varied with stability [table S2, permutational multivariate analysis of variance (PERMANOVA): *F* = 40.583, *R*^2^ = 0.032, *P* = 0.001], and pairwise compositional dissimilarity increased with stability differences among plots (table S2; Mantel: ρ = 0.055, *P* = 0.001). β Diversity decomposition indicated that this relationship was driven by abundance gradients (table S2; ρ = 0.088, *P* = 0.001) rather than the balanced-variation component (table S2; ρ = −0.026, *P* > 0.05). At the adult stage, PERMANOVA detected a statistically significant but small compositional shift with stability (table S2; *F* = 7.601, *R*^2^ = 0.006, *P* = 0.001), whereas Mantel tests for total β diversity and its components were not significant (table S2; *P* > 0.05).

Because understory light conditions may covary with seedling diversity, we additionally examined whether a light-related overstory structure proxy (adult basal area) was associated with seedling diversity and stability metrics. Using adult basal area within each 10 m–by–10 m seedling subplot as a light-related overstory structure index, we found that log-transformed adult basal area (hereafter, adult basal area) was positively associated with seedling species diversity and richness and negatively associated with seedling evenness (fig. S2, D to F), whereas adult basal area showed no significant relationship with seedling community stability or population stability (and only a weak association with asynchrony) (fig. S2, A to C). These results suggest that local overstory structure covaries with seedling diversity components but does not translate into detectable differences in seedling stability in our dataset.

### The effects of soil nutrients on diversity and temporal stability across life stages

**Fig. 3.**
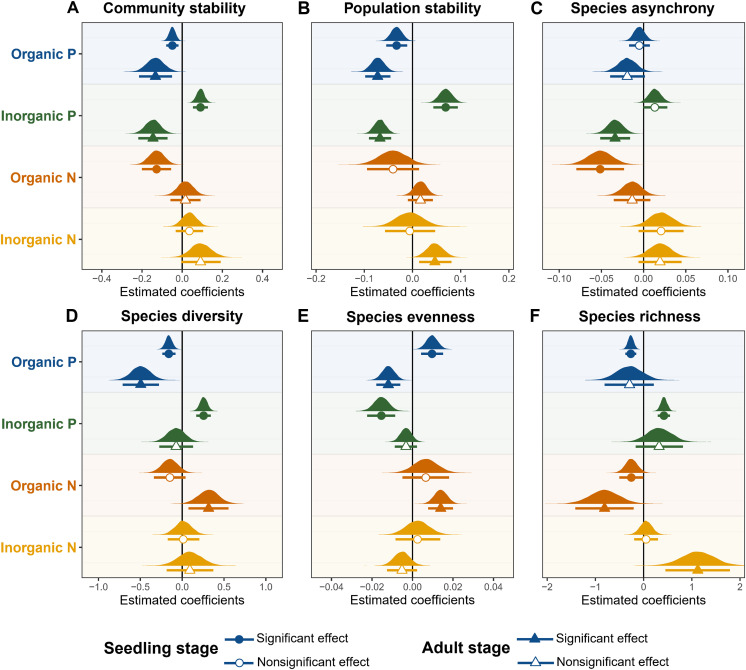
The effects of soil nutrients (different chemical forms of N and P) on diversity and stability across life stages. (**A** to **F**) show the effects on community stability (A), population stability (B), species asynchrony (C), species diversity (D), species evenness (E), and species richness (F). Different colors represent different soil nutrient predictors. Circles and lines show the estimated coefficients and 95% confidence intervals for the seedling stage, while triangles and lines represent the estimated coefficients and 95% confidence intervals for the adult stage. Coefficients were estimated from multiple linear regression models with all predictor variables centered and scaled. Parameter distributions and 95% confidence intervals were obtained using a nonparametric bootstrap with 10,000 replications. Filled symbols indicate significant effects (*P* < 0.05), while empty symbols indicate nonsignificant effects. Detailed statistical parameters are provided in table S3.

Soil organic P concentration was consistently negatively correlated with community stability (Fig. 3A and table S3; estimate = −0.049, *t* = −3.248, *P* = 0.001 for seedlings; estimate = −0.133, *t* = −3.330, *P* = 0.001 for adults) and population stability (Fig. 3B and table S3; estimate = −0.033, *t* = −2.953, *P* = 0.003 for seedlings; estimate = −0.072, *t* = −5.607, *P* < 0.001 for adults), with a marginally significant negative correlation with adult species asynchrony (Fig. 3C and table S3; estimate = −0.019, *t* = −1.880, *P* = 0.060). Inorganic P displayed a positive relationship with seedling community stability (Fig. 3A and table S3; estimate = 0.092, *t* = 5.465, *P* < 0.001), population stability (Fig. 3B and table S3; estimate = 0.069, *t* = 5.596, *P* < 0.001), and species asynchrony (Fig. 3C and table S3; estimate = 0.013, *t* = 2.079, *P* = 0.038), while showing a contrasting effect on adult stability (Fig. 3, A to C, and table S3). Organic N negatively affected seedling community stability and species asynchrony but did not influence adult stability (Fig. 3, A to C, and table S3). Inorganic N showed no significant correlations with seedling or adult community stability and species asynchrony (Fig. 3) except for positive effects on adult population stability (Fig. 3B and table S3; estimate = 0.047, *t* = 2.878, *P* = 0.004) and adult species richness (Fig. 3E and table S3; estimate = 1.127, *t* = 3.168, *P* = 0.002).

Higher soil organic P concentration was significantly associated with lower seedling species diversity (Fig. 3D and table S3; estimate = −0.160, *t* = −3.933, *P* < 0.001) and richness (Fig. 3F and table S3; estimate = −0.265, *t* = −4.740, *P* < 0.001), while it also showed significant negative associations with adult tree species diversity (Fig. 3D and table S3; estimate = −0.494, *t* = −4.359, *P* < 0.001) and evenness (Fig. 3D and table S3; estimate = −0.012, *t* = −4.024, *P* < 0.001). In contrast, soil inorganic P exhibited positive associations with seedling species diversity (Fig. 3D and table S3; estimate = 0.257, *t* = 5.682, *P* < 0.001) and richness (Fig. 3F and table S3; estimate = 0.421, *t* = 6.768, *P* < 0.001) but had no significant effects on adult tree species diversity, richness, and evenness. Organic N showed positive associations with adult tree species diversity (Fig. 3D and table S3; estimate = 0.314, *t* = 2.684, *P* = 0.007) and species evenness (Fig. 3E and table S3; estimate = 0.014, *t* = 4.546, *P* < 0.001) while having a slight effect on seedling richness (Fig. 3F and table S3; estimate = −0.253, *t* = −1.902, *P* = 0.057). Inorganic N showed no significant associations with seedling species diversity but was positively associated with adult tree species richness (Fig. 3F and table S3; estimate = 1.127, *t* = 3.168, *P* = 0.002). Overall, soil organic P showed consistent negative effects on diversity and stability across different life stages, while the effects of inorganic P varied depending on the life stage. These results suggest that soil P fractions played a more prominent role in shaping diversity and stability than soil N fractions.

### Predicting drivers of community stability using structural equation modeling

**Fig. 4.**
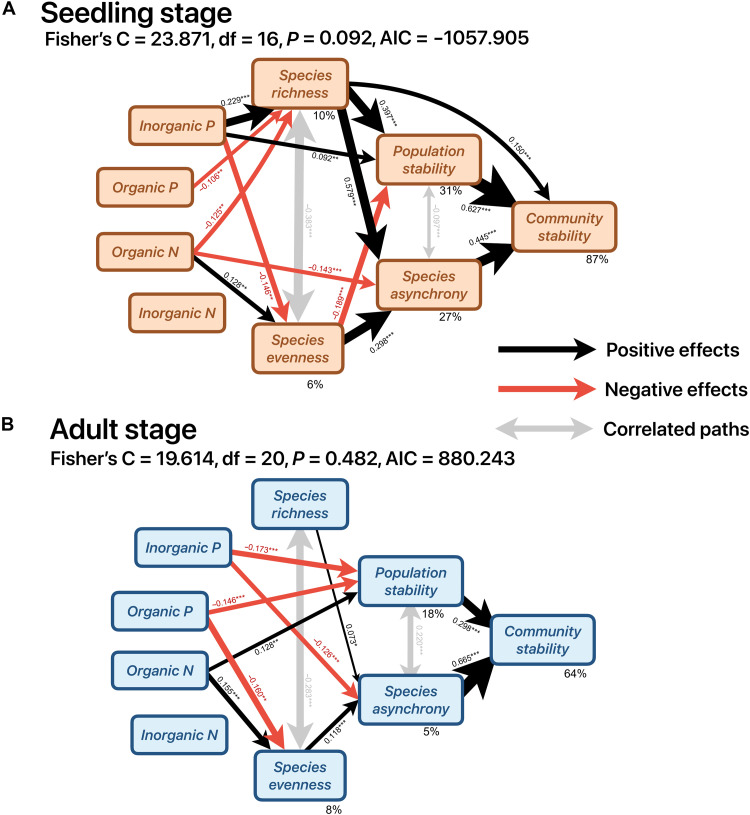
Results of the structure equation modeling. The structure equation modeling shows the causal paths of soil nutrients (different chemical forms of N and P) on species diversity (species richness and evenness), temporal stability (population and community stability), and species asynchrony for the seedling stage (**A**) and adult stage (**B**). Black and red arrows indicate positive and negative effects, respectively (*P < 0.05, **P < 0.01, ***P < 0.001). Gray arrows indicate correlated paths. Numbers along the arrows represent standardized path coefficients, and arrow size was positively proportional to the corresponding standardized path coefficients. For pSEM of the adult stage, the *R*^2^ corresponding to each response variable is obtained by spatial error models. For the pSEM of the seedling stage, the marginal *R*^2^ from linear mixed models is shown next to the response variables.

We used structural equation models to elucidate how soil nutrients drive diversity and stability across life stages. The global model fitted well (Fig. 4A, Fisher’s C = 23.871, df = 16, *P* = 0.092; Fig. 4B, Fisher’s C = 19.614, df = 20, *P* = 0.482) and showed that seedling richness mainly contributed to community stability (Fig. 4A; standardized total effects = 0.657) through both asynchrony and population stability, while seedling evenness displayed a minor effect on community stability (Fig. 4A; standardized total effect was 0.014). Adult tree species evenness was more strongly associated with community stability than adult tree species richness (Fig. 4B; standardized total effects were 0.078 and 0.049, respectively). This finding highlights the distinct mechanisms through which diversity at different life stages contributed to community stability.

At the seedling stage, higher soil inorganic P concentrations promoted community stability (Fig. 4A; standardized total effect was 0.207) through increasing seedling richness, population stability, and species asynchrony, whereas higher organic P and organic N concentrations weakened community stability (Fig. 4A; standardized total effects were −0.070 and −0.144, respectively) via reducing seedling richness. At the adult stage, inorganic P weakened community stability (Fig. 4B; standardized total effect was −0.135) through reducing species asynchrony and population stability, while organic P weakened community stability (Fig. 4B; standardized total effect was −0.056) via reducing adult tree species evenness and population stability (Fig. 4B). Higher organic N promoted community stability (Fig. 4B; standardized total effect was 0.050) through its positive effects on adult tree species evenness and population stability, whereas inorganic N was not linked to diversity and stability across both life stages (Fig. 4). Our structural equation model results indicated that soil P availability mainly influenced community diversity and stability across life stages: Inorganic P promoted diversity and stability at the seedling stage but reduced stability at the adult stage; organic P was negatively associated with both diversity and stability at the seedling and adult stages (the effect was more apparent at the adult stage because of its direct link to population stability; Fig. 4B).

### Robustness checks for temporal mismatch, temporal resolution, spatial confounding, and kriging uncertainty

To assess whether key nutrient-diversity-stability relationships were sensitive to temporal mismatch between snapshot soil surveys and long demographic series, differences in temporal resolution across life stages, and kriging prediction uncertainty, we conducted four robustness checks (Materials and Methods). Overall, these checks supported the robustness of our qualitative conclusions.

#### Directionality and temporal mismatch (seedlings and adults)

For seedlings, recomputing diversity and stability metrics within the temporally aligned window matching the soil survey (2017 to 2023) yielded regression effect directions consistent with the full-period analysis. As expected for a shorter time series, coefficient magnitudes and uncertainty differed to some extent, but no key conclusions were reversed (fig. S3).

In reverse-direction analyses treating nutrient forms as response variables and adult vegetation–related variables as predictors, apparent associations in nonspatial ordinary least squares (OLS) models were greatly reduced after accounting for spatial autocorrelation using spatial autoregressive (SAR) error models. Spatial dependence was strong across nutrient forms (λ = 0.977 to 0.998), and incorporating the spatial error term markedly improved model fit (ΔAIC = 2044 to 3287; AIC, Akaike information criterion) while reducing residual variance by 85 to 95% (table S6). In the SAR error models, few vegetation predictors remained statistically significant and effect sizes were small (table S7), suggesting that nutrient heterogeneity is dominated by persistent shared spatial gradients, with comparatively weak detectable vegetation-to-soil feedback signals.

#### Temporal-interval sensitivity (seedlings)

To evaluate whether seedling-stage inferences depend on annual sampling frequency, we recalculated seedling productivity and stability metrics using nonoverlapping 3-, 4-, and 5-year census intervals and refitted the piecewise structural equation models (pSEMs). Across these temporal resolutions, the overall pSEM structure and the directions of major pathways were qualitatively consistent with the annual analysis, and model fit remained acceptable (Fisher’s C tests, *P* > 0.05 in all panels; fig. S4). The explained variance in community stability also remained high across interval definitions (fig. S4, *R*^2^ = 0.87, 0.86, 0.83, and 0.65 for annual, 3-, 4-, and 5-year intervals, respectively). As expected, some coefficient magnitudes and uncertainty varied under coarser intervals due to fewer effective time points, but no key pathways were reversed.

#### Kriging uncertainty propagation

To assess the robustness of our main inferences to uncertainty in kriged soil nutrients, we propagated quadrat-level kriging prediction variance into downstream regressions and the pSEM using Monte Carlo sampling. Across Monte Carlo realizations, the directions of key nutrient-related regression coefficients and major pSEM pathways were consistent with the main analysis for both the seedling stage (fig. S5) and the adult stage (fig. S6). As expected, incorporating kriging uncertainty modestly increased uncertainty around some estimates and slightly shifted a subset of coefficient magnitudes, but the qualitative interpretation of nutrient-diversity-stability linkages remained unchanged.

### Results of neighborhood models

**Fig. 5.**
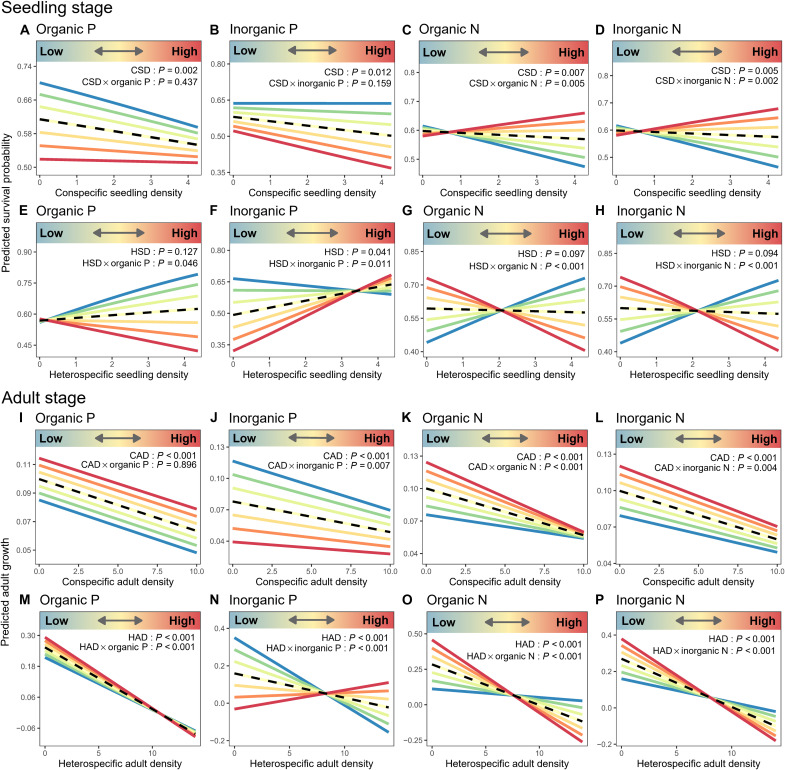
Nutrient form–dependent conspecific and heterospecific neighborhood effects across life stages. (**A** to **H**) show the results of seedling stage, and (**I** to **P**) show the results of adult stage. Colored lines depict the fitted relationships between individual performance (seedling survival rate or adult DBH growth) and conspecific and heterospecific neighbor density under varying soil nutrient levels (refer to the color bar in each panel). The four columns from left to right represent organic P, inorganic P, organic N, and inorganic N, showing how soil nutrient levels modify density-dependent effects. The black dashed line represents predictions at the average soil nutrient level. Significant interaction terms indicate that conspecific density effects (intraspecific competition) and heterospecific density effects (interspecific competition) are modulated by soil nutrients. From left to right, the nutrients are organic P, inorganic P, organic N, and inorganic N. CSD, HSD, CAD, and HAD are the abbreviations for conspecific seedling density, heterospecific seedling density, conspecific adult density, and heterospecific adult density, respectively. Detailed statistical parameters are provided in tables S4 and S5.

To mechanistically interpret the community-level pathways identified by pSEM (Fig. 4), we fitted neighborhood models to test whether nutrient forms modify conspecific versus heterospecific neighborhood effects on seedling survival and adult growth across life stages (Fig. 5). In the seedling survival models, we observed negative effects of conspecific seedling density (CSD) on seedling survival (Fig. 5, A to D, and table S4, main effects), while heterospecific seedling density (HSD) showed neutral or positive effects on seedling survival (Fig. 5, E to H, and table S4, main effects). The effect of HSD on seedling survival ranged from negative (strengthened interspecific competition) at high values of organic P to positive (strengthened interspecific facilitation) at low values of organic P (Fig. 5E, HSD × organic P: *P* = 0.046), while the positive effect of HSD on seedling survival was enhanced at high values of inorganic P (enhanced interspecific facilitation) (Fig. 5F, HSD × inorganic P: *P* = 0.011). Increasing values of organic and inorganic P reduced seedling survival, but they did not interact with conspecific density (Fig. 5A, CSD × organic P: *P* = 0.437; Fig. 5B, CSD × inorganic P: *P* = 0.159). In addition, increasing values of both organic N and inorganic N reversed the sign of the response to heterospecific density from positive, indicating strengthened interspecific facilitation at low N values, to negative, indicating strengthened interspecific competition at high N values (table S4, estimates for the interaction terms = −0.112 and −0.104, both *P* < 0.001, respectively). Increasing values of organic and inorganic N displayed the opposite interaction with conspecific density to those described for heterospecific density, with lower values of both N fractions strengthening intraspecific competition (table S4, estimates for the interaction terms with conspecific density = 0.065 and 0.068, *P* = 0.005 and 0.002, respectively). Results of seedling neighborhood models indicated that soil nutrients altered seedling density in six of the eight cases: Interspecific competition was strengthened in plots containing higher concentrations of soil organic P, organic N, and inorganic N, while intraspecific competition was enhanced in plots with lower concentrations of both soil N fractions. Furthermore, there was evidence of facilitation among species on plots with low values of organic P, organic N, and inorganic N, and those with high values of inorganic P, and facilitation within species on plots with high values of organic N and inorganic N.

In the adult tree growth model, increasing conspecific (Fig. 5, I to L, and table S5) and heterospecific neighborhood densities (Fig. 5, M to P, and table S5) both significantly reduced tree growth, indicating the occurrence of CNDD and heterospecific negative density dependence (HNDD). Furthermore, increasing organic P exacerbated the negative effects of heterospecific adult density (HAD; Fig. 5M, HAD × organic P: *P* < 0.001) without significantly affecting the response to conspecific adult density (CAD; Fig. 5I, CAD × organic P: *P* = 0.896). Higher inorganic P concentrations mitigated both intraspecific (Fig. 5J, CAD × inorganic P: *P* = 0.007) and interspecific competition (Fig. 5N, HAD × inorganic P: *P* < 0.001), with a stronger effect on interspecific competition (table S5, *t* = 24.852 and 2.686, respectively). Notably, the effect of HAD on adult growth shifted from negative at low values of inorganic P to neutral at high values of inorganic P (Fig. 5N). Organic and inorganic N predominantly intensified interspecific competition (table S5, *t* = −31.427 and −27.841, respectively) despite minor alleviation of intraspecific competition (table S5, *t* = −4.177 and −2.863, respectively). Combined with previous results of seedling neighborhood models, we found that higher organic P concentrations consistently intensified interspecific competition across life stages, whereas higher inorganic P concentrations alleviated interspecific competition. Both organic and inorganic N exacerbated interspecific competition across life stages.

## DISCUSSION

Most previous studies on forest stability have concentrated on a single life stage, leaving open the question of whether the drivers of stability differ across stages. Yet, forest communities are structured by a continuum of life stages, from seedlings that underpin recruitment and regeneration (*19*, *53*) to adults that dominate carbon storage and ecosystem functioning (*20*). Understanding how stability is maintained across life stages is crucial for sustaining forest ecosystem functions and services under climate change. By explicitly integrating decade-scale seedling and adult demographic data, covering 31,636 seedlings and148,845 adults, with high-resolution soil nutrient mapping, our study provides a rare opportunity to test how life-stage-specific diversity components and nutrient dynamics jointly regulate the temporal stability of subtropical forest communities.

Our study reveals a consistent positive relationship between diversity and stability (Figs. 1 and 2), with species richness driving seedling stability and species evenness driving adult stability. This finding highlights the often-overlooked importance of evenness (*32*, *54*), which can be as crucial as richness in maintaining the stability of natural forest ecosystems. Furthermore, by integrating soil nutrient mapping and neighborhood models, we found that soil P availability mediates these relationships through stage-specific mechanisms. Inorganic P enhanced seedling stability by alleviating competition and promoting diversity but reduced adult stability by suppressing species asynchrony. In contrast, organic P consistently reduced diversity and stability across both stages by intensifying interspecific competition. Soil N exerted comparatively weaker effects. Collectively, these findings highlight the pivotal role of soil P availability in regulating life stage–specific stability in P-limited subtropical forests, emphasizing the need to incorporate ontogenetic shifts in nutrient use and differential effects of P forms into forest conservation and management strategies.

### The diversity-stability relationships across life stages in a subtropical forest

Our study uncovers life stage–specific diversity-stability relationships in a subtropical natural forest. At the seedling stage, seedling species richness showed the strongest association with community temporal stability (table S1). Greater species richness reflects higher temporal and spatial niche differentiation among coexisting species (*55*), which enhances species asynchrony (*5*) and enables communities to buffer climatic and environmental disturbances, thereby increasing stability. This finding is consistent with previous studies from grasslands (*25*, *56*) and forest communities (*17*), where species richness has been shown to strengthen ecosystem stability.

In contrast, at the adult stage, adult tree species evenness, rather than adult tree species richness, was more strongly associated with community stability (table S1). This pattern likely stems from the highly uneven species abundance distributions in natural forests (*36*, *57*, *58*). Asymmetric competitive abilities and strategies among tree species, coupled with prolonged species interactions and environmental filtering, often lead to communities dominated by a few abundant species, with most others remaining rare (*58*). Dominant species contribute disproportionately to community productivity and stability according to the mass ratio hypothesis (*59*), whereas rare species, despite their numerical presence, have a limited impact. This pattern aligns with recent global findings that the positive effects of species richness on ecosystem net primary productivity often weaken or disappear in ecosystems with low evenness, particularly in moist tropical and subtropical forests (*36*). Such forests, including our study system, typically exhibit a “many-rare–species, few-abundant–species” structure shaped by long-term ecological interactions (*57*). Consequently, increasing species richness in such systems was not consistently associated with higher stability. Instead, greater evenness reduces the dominance of dominant species and was associated with greater stability (*32*). These findings underscore the importance of recognizing life stage–specific diversity-stability relationships in natural forest ecosystems. The distinct roles of species richness and evenness, particularly the critical role of evenness in later life stages, highlight the complementary contributions of rare and dominant species to ecosystem functioning and stability. Within the spatial scale and productivity-based definition of stability used here, greater adult evenness (i.e., reduced dominance and higher relative abundance of less common species) was associated with higher temporal stability, highlighting evenness as an important component of stability maintenance in natural forests.

We further examined whether quadrats differing in community stability also differ in community composition (table S2). At the seedling stage, community composition changed along the stability gradient, and quadrats that differed more in stability were also more dissimilar in composition (table S2). β-Diversity decomposition indicated that this pattern was driven mainly by abundance gradients (differences in dominance structure) rather than balanced variation (table S2), suggesting that more versus less stable seedling quadrats differ primarily in relative dominance patterns among largely shared species pools rather than species replacement. At the adult stage, compositional differences along the stability gradient were present but weak, and pairwise stability differences did not translate into a consistent increase in pairwise compositional dissimilarity (table S2). Overall, these results indicate that stability differences are accompanied by clearer shifts in community dominance structure at the seedling stage than at the adult stage.

### Soil P availability regulated community stability through its effects on interspecific competition and diversity

The increased atmospheric N deposition in subtropical forests has been linked to intensified P limitation (*40*), a key factor influencing tree growth and community dynamics. While extensive research in grassland ecosystems has examined the consequences of P enrichment on diversity (*60*, *61*) and ecosystem stability (*15*, *62*, *63*), our understanding of forest ecosystems’ responses remains limited. Our study demonstrates that the effects of soil P on community stability vary depending on the life history stage and the form of P. Specifically, we found that soil inorganic P was associated with higher community stability at the seedling stage but lower stability at the adult stage (Fig. 3A), indicating that inorganic P may regulate community stability through distinct mechanisms at different life stages. The observed variation in soil P among quadrats within the plot may reflect long-term pedogenic and within-plot (hillslope-scale) processes in subtropical forests, including variation in weathering intensity and in microtopography and slope position that drives topographic redistribution along erosion-deposition and hydrological flow paths (*42*). Differences in mineral-associated P retention (e.g., adsorption and partial occlusion) and local biological recycling may further contribute (*42*, *64*). Although we cannot quantify the relative contributions of these processes with the current dataset, this context supports interpreting soil P as a relatively persistent edaphic template that shapes local competitive environments and thereby contributes to diversity and stability patterns across life stages.

At the seedling stage, increased soil inorganic P alleviated interspecific competition (Fig. 5F) for limited P resources, promoting seedling species coexistence. This enhanced seedling species richness mediated community stability under high inorganic P conditions through greater asynchrony and population stability (Fig. 4A). In contrast, at the adult stage, the decline in asynchrony mediated by inorganic P (Fig. 3C) was the dominant driving factor of reduced community stability (Fig. 4B), supporting observations from grassland systems (*63*, *65*). Higher inorganic P eliminated interspecific competition among adults (Fig. 5N, individual growth was nearly independent of heterospecific neighbor density under high inorganic P), contributing to the loss of compensatory dynamics among species. In addition, enriched inorganic P reduces the temporal and spatial differentiation of P resources among tree species (*44*, *46*, *47*), leading to a convergence in resource utilization. Such convergence limits differential species responses to environmental fluctuations, thereby reducing asynchrony and undermining community stability.

This reversal in inorganic P effects between life stages reflects a fundamental ontogenetic shift in resource dependency. Seedlings can buffer competition by relying on seed-stored resources (*66*, *67*) benefit from competition alleviation under high inorganic P, facilitating establishment and coexistence. Adults, dependent more on soil-derived nutrients, experience a suppression of compensatory dynamics when interspecific competition is reduced under high inorganic P (Fig. 5N), which diminishes asynchrony and overall stability. Therefore, the changes in soil inorganic P can produce contrasting effects on community stability depending on tree life stage, emphasizing the importance of considering life history stage when evaluating the mechanisms linking soil nutrient availability, species interactions, and forest community stability.

Past work has demonstrated pervasive resource partitioning for various P forms among tree species in subtropical forests (*44*), and so we separated the contributions of soil inorganic and organic P to the long-term community dynamics across life stages. In contrast to the association of higher inorganic P with an alleviating of interspecific competition, we found that higher organic P was consistently associated with intensified interspecific competition, reduced species diversity, and lower community stability across life stages, which is in line with our hypothesis that a greater organic P pool may destabilize community stability. Soil microbes, particularly ectomycorrhizal (ECM) fungi, may mediate the relationship between soil organic P and community stability (*21*). ECM trees gain a competitive advantage through phosphatase enzymes secreted by their ECM fungal partners, potentially enhancing the mobilization of phosphate from organic-associated P pool (*68*). In addition, ECM fungi provide protection to roots and facilitate resource transfer (*69*), promoting a positive plant-soil feedback (*70*). This feedback can intensify interspecific competition, leading to the formation of low-diversity, monodominant communities over multiple generations (*21*, *71*). Our findings suggest that changes in mycorrhizal dominance and species diversity along the soil organic P gradient may mediate the alteration of community stability across life stages.

### Soil N availability showed weak effects on diversity and stability

Atmospheric N deposition has become a significant threat to biodiversity and ecosystem stability since the onset of the industrial era (*14*, *72*). While its adverse effects, including the loss of plant diversity (*2*) and reduced species asynchrony and ecosystem stability (*14*), have been extensively documented in grasslands, much less is known about how N availability influences diversity and stability in forest communities, particularly across different life stages of trees. Our findings indicate that higher soil organic N concentrations were associated with lower species richness (Fig. 3F) and intensified interspecific competition across life stages (Fig. 5, G and O), aligning with previous research (*38*, *73*). In contrast, spatial variation in soil inorganic N was not related to species richness at either life stage (Fig. 4, A and B).

In terms of temporal stability, both organic and inorganic N showed overall weaker effects on community stability than P fractions (Fig. 4, A and B), likely due to N saturation in our subtropical forest system, where soil total N concentrations are already high from long-term atmospheric inputs. Notably, the effects of organic N on community stability varied by life stage: It tended to reduce stability at the seedling stage but slightly enhanced it at the adult stage. This life stage–dependent response may reflect ontogenetic shifts in nutrient demand and foraging capacity, as adult trees typically require and acquire more nutrients than seedlings (*23*). In contrast, soil inorganic N was not significantly associated with community stability at either life stage. Together, these findings underscore the importance of considering both the chemical form of N and the ontogenetic stage of trees when assessing the ecological implications of N availability in forest ecosystems.

Despite these advances, several limitations should be noted. First, soil nutrient concentrations were measured only once, providing a static snapshot rather than temporal dynamics; this limitation is partly offset by the high spatial resolution of our sampling design, but repeated soil measurements would be required to directly quantify temporal variability. Because tree censuses span multiple years, we interpret soil nutrients primarily as relatively persistent spatial gradients over the census period. To evaluate potential temporal mismatch and reverse-causality concerns, we conducted sensitivity analyses. For the adult stage, reverse-direction analyses that treated nutrient forms as response variables and vegetation-related variables as predictors showed that spatial dependence was strong and that apparent associations were largely attenuated once spatial autocorrelation was accounted for in SAR error models (tables S6 and S7). For seedlings, using soil nutrients measured in 2017 to predict seedling diversity and stability over 2017 to 2023 produced qualitatively similar results to the main analyses (fig. S3), supporting the robustness of our inferences to temporal alignment. In addition, because kriged nutrient concentrations are model-based predictions with associated uncertainty, we propagated quadrat-level kriging prediction variance into downstream regressions and the pSEM via Monte Carlo sampling, and the main conclusions were robust to this uncertainty (figs. S5 and S6). Second, stability estimates differ in temporal resolution across life stages: Seedling stability is derived from denser annual observations, whereas adult stability is estimated from fewer, multiyear censuses, which may affect precision more than the qualitative direction of life-stage contrasts. To address this difference in temporal resolution, we conducted an additional sensitivity analysis for seedlings by recalculating seedling stability (and diversity-related metrics) using coarser temporal intervals of 3-, 4-, and 5-year windows. Across these alternative calculations, the directions of key relationships and the qualitative conclusions remained consistent with the main results (fig. S4), indicating that our seedling-stage inferences are robust to choices of temporal interval. Third, our P metrics are based on the standards, measurements and testing (SMT) operational fractionation: SMT-inorganic P may include a range of mineral-associated forms (including less readily available and partially occluded fractions), and SMT-organic P is calculated as a residual pool (total P minus inorganic P) that integrates multiple components. Accordingly, our results should be interpreted as responses to spatial gradients in these operational pools rather than direct measures of immediate P bioavailability or specific acquisition pathways; any occluded component in SMT-inorganic P would mainly add noise and thus tend to attenuate biotic associations, making our estimates conservative. Last, although we integrated demographic and nutrient data, belowground processes such as mycorrhizal associations, phosphatase activity, and microbial-mediated P cycling were not directly measured, constraining our ability to disentangle their mechanistic roles in regulating community stability. Future work combining repeated soil monitoring, lability-resolved P fractions, and direct assessments of plant-microbe interactions will be essential to test the robustness and generality of our conclusions across broader spatial and temporal contexts.

This study reveals how the mechanisms underlying subtropical forest community stability shift across life stages: The diversity-stability relationship shows a clear stage-dependent pattern—Seedling community stability is driven primarily by species richness, while adult tree community stability depends mainly on species evenness. These findings update previous theoretical understandings based on single life-history stages and highlight the importance of adopting a life-stage perspective when interpreting stability patterns. Our inference is restricted to productivity-based temporal stability at the small scale over a decadal-scale observation window. Within this defined scale and metric, our results suggest that maintaining seedling richness and avoiding excessive adult tree dominance (higher adult tree evenness) was associated with greater temporal stability, whereas whether and how these relationships scale up to broader ecosystem resilience across functions and larger spatial extents remains to be tested. On the other hand, this study highlights that the impact of P availability on forest stability has often been oversimplified. Traditional studies based on single P forms or single life-history stages may have misestimated forest community responses to global change. By distinguishing inorganic and organic P pools, we show that different P forms are associated with different pathways through species interactions, asynchrony, and temporal stability. Incorporating P forms and their potential links to key belowground functional groups, such as mycorrhizal fungi, may help improve mechanistic understanding of forest response to global change.

## MATERIALS AND METHODS

### Data collection of tree growth dynamics

We performed this study in the 50-ha permanent forest dynamics plot in the Heishiding Nature Reserve in southern China. This site (111°49′ E–111°55′ E, 23°25′ N–23°30′ N, 150- to 927-m altitude) belongs to a subtropical evergreen broad-leaved forest and has a subtropical moist monsoon climate, with a mean annual temperature of 19.6°C and average annual precipitation of 1744 mm. We used the field survey data of the Heishiding 50-ha permanent forest plot to quantify forest community structure and productivity. Within the 50-ha plot, all woody stems with a diameter at breast height (DBH) ≥1 cm have been tagged, mapped, identified, and measured every 5 years since 2011. We defined adults as trees with DBH ≥1 cm and seedlings as individuals with DBH <1 cm. Seedlings that reached 1-cm DBH during census intervals were excluded from seedling-stage analyses. To investigate seedling dynamics, six 1-ha plots were established adjacent to the 50-ha plot in 2008, with three plots located at high altitude (600 m) and the remaining three at low altitude (340 m). The altitude had no effect on species composition (*74*). Each 1-ha plot was divided into 100 subplots (10 m by 10 m). Seedling quadrats were placed in alternating subplots (50 per plot), with four 1 m–by–1 m quadrats in each selected subplot (see fig. S8 for the quadrat arrangement). Thus, each 1-ha plot included 200 seedling quadrats (50 by 4), yielding a total of 1200 1 m–by–1 m seedling quadrats across the six plots. All seedlings of woody plants with DBH less than 1 cm have been tagged, mapped, identified, and censused annually for height from 2008 to 2023. Across these plots, we recorded 402 tree species from 190 genera and 80 families, covering 31,636 seedlings and 148,845 adults.

### Field soil sampling

To determine soil nutrient concentrations, we collected 1288 soil samples across the 50-ha plot in July 2023 and 600 soil samples across the six 1-ha plots in July 2017 (*9*), respectively. In the 50-ha plot, we collected one soil sample in every alternate 20 m–by–20 m quadrat, resulting in 588 regular soil samples. We randomly selected two regular sample points within each 100 m–by–100 m plot to capture directional variation in soil properties. For each point, we collected seven soil samples at distances of 0.16, 0.33, 0.61, 1.03, 2.66, 6.22, and 7.67 m away from these regular sites along a randomly chosen diagonal direction, to capture spatial distribution and variation of soil properties at fine scales, resulting in 700 cluster soil samples (see fig. S7 for details of sampling design). In addition, the sampling design in the six 1-ha plots followed a similar approach (see fig. S8 for details of sampling design). At each sampling point, we collected a 50-g soil sample (0- to 10-cm depth), which was then flash-frozen on dry ice and stored at −20°C for subsequent physicochemical analyses. In the laboratory, soil samples were sieved through a 2-mm mesh to remove stones and roots. After sieving, each sample was partitioned into subsamples for nutrient analyses and then stored at −20°C.

Soil total N was initially digested by concentrated sulfuric acid, and inorganic N (ammonium, nitrite, and nitrate) was extracted with 1 M potassium chloride using the indophenol-blue colorimetric method, and their concentrations were measured with a BluVision discrete analyzer (BluVision; Skalar, Breda, Holland). Soil total P and inorganic P were extracted following the SMT protocol (*75*) and also measured on the same analyzer. Soil organic N (or P) was determined by subtracting inorganic N (or P) from the soil total N (or P) concentrations. Under these operational definitions, organic N and organic P represent the difference-based (residual) pools derived from total and inorganic measurements and are used here as operational indicators of soil N and P partitioning. The spatial prediction of soil nutrient concentrations for all quadrats was obtained by kriging interpolation (see text S1 for details) conducted using the R package gstat (*76*).

### Calculation of diversity

Species richness (S) is defined as the total number of tree species in a quadrat. We used Hill’s evenness index to estimate species evenness (*31*). By definition

$$\text{Species evenness}=\frac{e^{H}}{S}$$ (1)

where H denotes Shannon’s entropy and S is the species richness. Species evenness approaches its maximum value of 1 in communities composed of equally abundant species and approaches a minimum value of 0 in communities dominated by a few species. Shannon’s entropy was quantified as $\sum_{i=1}^{S}p_{i}\text{ln}\left(p_{i}\right)$ for each quadrat. For adult trees, $p_{i}$ represents the proportion of basal area ($\text{BA}=\frac{\pi }{4}\times \text{DB}H^{2}$) contributed by species *i*, while for seedlings, $p_{i}$ is the proportion of the number of individuals belonging to species *i*. Basal-area weighting was used for adults because it better captures biomass dominance and the uneven occupation of space among species, and avoids overweighting the many small stems included in the DBH ≥1 cm census; abundance weighting was used for seedlings because basal area was unavailable for DBH <1 cm individuals.

Considering that species richness alone cannot fully capture species diversity in a highly uneven community (*58*), we also quantified species diversity using the effective species number (*31*, *77*)

$$\text{Species diversity}=S\times \frac{e^{H}}{S}=e^{H}$$ (2)

In communities with greater evenness, the effective number of species approaches the species richness more closely.

### Calculation of productivity and temporal stability

For adult trees, we estimated the AGB using a locally derived DBH-based allometric equation (text S2), and adult tree aboveground productivity was calculated as the increment in AGB between consecutive censuses. For seedlings, DBH-based allometry is not applicable and only height was measured annually; therefore, we used height increment as a proxy of aboveground seedling productivity. For each nonoverlapping 20 m–by–20 m adult quadrat and 1 m–by–1 m seedling quadrat, productivity for each species was quantified as the sum of individual productivity of all conspecific living individuals (including newly recruited individuals) and community productivity as the sum of productivity across all species. For newly recruited individuals, productivity was calculated as their AGB (adults) or height (seedlings) at the census when they were first recorded (assuming zero at the start of the interval).

Community-level temporal stability (community stability) was defined as the inverse of the coefficient of variation of community productivity through time and was calculated as $\frac{\mu_{\text{comm}}}{\sigma_{\text{comm}}}$, where $\mu_{\text{comm}}$ and $\sigma_{\text{comm}}$ denote the temporal mean and SD of community productivity, respectively. Species-level temporal stability (here defined as population stability) (*9*, *26*), reflecting the mean level of invariability in the productivity of individual species, is measured as the inverse of the abundance-weighted coefficient of variation of the productivity of a species through time (*27*) and is calculated as $\frac{\mu_{\text{comm}}}{\sum_{i=1}^{n}\sigma_{sp_{i}}}$, where $\sigma_{sp_{i}}$ is the temporal SD of annual productivity of species i in a quadrat with n species. To quantify the asynchrony in population productivity dynamics, we use the community-wide metric of species asynchrony $1-\frac{\sigma_{\text{comm}}}{{\left(\sum_{i=1}^{n}\sigma_{sp_{i}}\right)}^{2}}$ (*5*, *78*). Species asynchrony ranges from 0 (absolutely synchronous population productivity dynamics) to 1 (absolutely asynchronous population productivity dynamics). An upward trend of productivity over time may mask the temporal variation in productivity (*17*), so we removed this effect by regressing both community and population productivity against census year and used the SD of the residuals to calculate temporal stability and asynchrony (*9*, *56*). Because seedlings and adults are monitored at different temporal resolutions (annual versus 5-year censuses), we do not interpret the absolute magnitude of stability as directly comparable across life stages; inference focuses on life stage–specific relationships estimated within each stage.

### Statistical analysis

For adult tree analyses, we divided the 50-ha plot into nonoverlapping 20 m–by–20 m quadrats, while seedling analyses were based on the 1200 permanently established 1 m–by–1 m quadrats. These quadrat sizes are aligned with the minimum resolution of our soil nutrient sampling design (20 m–by–20 m in the 50-ha plot and 1 m–by–1 m in the seedling plots, as described below), ensuring that vegetation and soil data are spatially comparable.

We first applied bivariate linear regression to test the hypothesized positive relationships among population stability, species asynchrony, and community stability. Multiple linear regression models were then used to evaluate the effects of different forms of soil nutrients on species diversity, community stability, and their components. Before analysis, all predictor variables were centered and scaled to allow direct comparison of regression coefficients. Because species asynchrony and evenness are bounded between 0 and 1, we additionally assessed the robustness of models using these variables as responses by refitting them with beta regression and a logit link [R package betareg (*79*)]. Coefficients are reported on the link (logit) scale. These beta-regression models were used as robustness checks, and the qualitative conclusions (effect directions and inference) were unchanged (table S8).

To further explore the pathways by which soil nutrients affect community stability, we constructed pSEMs using the piecewiseSEM package (*80*). The initial model structure followed a conceptual framework (fig. S9). In addition to these hypothesized mediation paths, we allowed ecologically plausible direct effects (e.g., nutrient forms on population stability and species asynchrony, richness/evenness on community stability) as candidate paths in the component models. We adopted a model selection approach to determine the final pathways. Specifically, for each response variable in the conceptual model, we fitted all possible submodels and selected the one that minimized the AIC. To ensure that collinearity among predictors did not bias coefficient estimates, we assessed multicollinearity for each component model using variance inflation factors and report the results in table S9. The overall fit of the final pSEM was evaluated using Shipley’s d-separation test of conditional independence (*81*) based on Fisher’s C statistic (*P* > 0.05 indicating an acceptable fit). All these analyses were conducted separately for the seedling and adult quadrats.

To address potential spatial dependencies when conducting regression analyses described above for adult quadrats (nonoverlapping 20 m–by–20 m quadrats), we applied an SAR error model using the spatialreg package (*82*), incorporating a queen contiguity spatial weights matrix, which considers each plot’s neighbors to include both its four edge-adjacent plots and its four corner-adjacent plots. For the seedling quadrat data, we used linear mixed-effects models using the lme4 package (*83*), with the 10 m–by–10 m subplots treated as random effects to account for potential topographic heterogeneity (e.g., elevation and slope) and the associated spatial correlations. To ensure the normality and homoscedasticity of the model residuals, community stability, population stability, and all soil nutrient variables were log-transformed before analysis.

Because light availability may influence seedling growth and diversity, we conducted an additional analysis to examine whether a light-related overstory structure gradient covaries with seedling diversity and stability patterns. Direct light measurements were not available across all seedling quadrats, so we used local adult basal area as a proxy for potential shading. Specifically, for each 10 m–by–10 m seedling subplot, we summed the basal area of all adult stems (DBH ≥1 cm) within the subplot and log-transformed adult basal area to reduce right-skewness. We then fitted separate linear models, relating log-transformed adult basal area to each seedling response variable, including diversity metrics (species richness, evenness, and diversity) and stability-related metrics (community stability, population stability, and species asynchrony).

To evaluate whether quadrats differing in community stability also differ in community composition, we quantified between-quadrat compositional dissimilarity (β diversity) using Bray-Curtis distances for both the seedling and adult stages. We tested compositional variation along the community stability gradient using PERMANOVA (999 permutations) and assessed whether pairwise compositional dissimilarity increases with pairwise differences in community stability using Mantel tests (999 permutations). We further decomposed Bray-Curtis β diversity into balanced variation in abundance ($\beta_{\text{BRA}}$) and abundance gradients ($\beta_{\text{GRA}}$) (*84*), where $\beta_{\text{BRA}}$ captures turnover-like changes driven by roughly compensatory substitutions among species (i.e., similar total abundance but redistributed among species), whereas $\beta_{\text{GRA}}$ captures dominance-structure differences arising from directional abundance gradients or nestedness-like patterns (i.e., one assemblage representing an abundance-reduced/enhanced subset of the other), to identify which component is most closely associated with stability differences. All statistical analyses were performed in R 4.2.2 (*85*).

### Sensitivity analyses

Because soil nutrients were measured once while demographic series span multiple years, and because kriged soil variables carry prediction uncertainty, we conducted four robustness checks. These checks address (i) directionality concerns arising from temporal mismatch and potential reverse-direction vegetation-soil feedbacks under strong spatial dependence, (ii) sensitivity to seedling temporal resolution, and (iii) uncertainty in kriged nutrient predictors.

### Directionality and temporal mismatch

#### Temporal alignment sensitivity (seedlings, 2017 to 2023)

To reduce potential bias arising from linking a single soil survey (July 2017 for seedling plots) to stability metrics computed over a much longer period (2008 to 2023), we recalculated all seedling-stage variables—community productivity, community stability, richness, evenness, population stability, and species asynchrony—using a temporally aligned window (2017 to 2023). Within this aligned window, stability components were computed using the same definitions as in the main analysis. We then refitted the key regression models using identical specifications and evaluated whether the direction and qualitative conclusions were maintained (fig. S3).

#### Reverse-direction models with spatial control (adults)

To evaluate the plausibility that adult vegetation properties and productivity dynamics could feedback to shape spatial patterns in soil nutrient forms (reverse causality), we conducted reverse-direction analyses in which each nutrient form was treated as a response variable, and adult vegetation-related variables (e.g., richness, evenness, species asynchrony, population stability, temporal mean productivity, and temporal SD of productivity) were treated as predictors. Because both soils and vegetation show strong spatial structure at the 20 m–by–20 m scale, we explicitly accounted for spatial autocorrelation by fitting (i) nonspatial OLS models and (ii) SAR error models using a queen-contiguity weights matrix based on 20 m–by–20 m quadrat adjacency. Predictors were standardized (z-transformed), and nutrients were transformed as in the main analysis. We compared OLS and SAR error results using standardized coefficients and spatial diagnostics (e.g., the spatial error parameter and information criteria) to assess whether vegetation predictors explained additional nutrient variation beyond dominant shared spatial gradients (tables S6 and S7).

### Temporal-interval sensitivity

To test whether seedling-stage inference depends on annual sampling frequency and to improve comparability with adult 5-year census intervals, we rederived seedling productivity time series using 3-, 4-, and 5-year nonoverlapping census intervals across 2008 to 2023. For each interval length, we recomputed seedling community stability using the same definition as in the main analysis. We then refitted the key regression models and pSEM using these interval-based stability metrics to assess whether the direction and qualitative interpretation of the main effects changed. Full results are reported in fig. S4.

### Kriging uncertainty propagation

Because kriged nutrient values are predictions with quadrat-specific uncertainty, we propagated kriging prediction variance into main regressions model and the pSEM via Monte Carlo sampling. For each nutrient and quadrat, kriging provides a predicted value $\hat{X}$ and a prediction variance $\sigma_{k}^{2}$. We generated repeated realizations (500 times) by drawing $X∼N\left(\hat{X},\sigma_{k}^{2}\right)$, refit all downstream models for each draw, and summarized inference from the resulting distributions of regression coefficients and SEM path estimates (median and 95% intervals). Full details and results are provided in figs. S5 and S6.

### Neighborhood model of seedling survival and adult growth

#### Aim, causal assumptions, and interpretation boundaries

Our neighborhood models are designed to quantify the strength and direction of local biotic interactions and to test whether these interaction effects vary along soil nutrient gradients. Accordingly, the primary estimands are (i) the main effects of conspecific and heterospecific neighborhood density, interpreted as the magnitude and direction of density dependence (CNDD and HNDD) and (ii) the nutrient × neighborhood interaction terms, interpreted as nutrient-dependent modification of neighborhood effects. These models are not intended to estimate the total causal effect of soil nutrients on individual survival or growth.

To make the underlying assumptions explicit and to avoid ambiguity in interpretation, we specified a directed acyclic graph (DAG; fig. S10) describing expected relationships among soil nutrients, neighborhood density, and individual performance (seedling survival and adult growth). In this framework, soil nutrients may influence individual performance directly and indirectly via changes in neighborhood structure, while neighborhood density affects performance by capturing local biotic interactions; nutrient availability may further modify the neighborhood-performance relationship, motivating nutrient × neighborhood interaction terms. Therefore, throughout this section, we interpret (a) soil nutrient main effects as effects conditional on neighborhood context (not total nutrient effects), (b) neighborhood main effects as neighborhood effects conditional on nutrient context, and (c) nutrient × neighborhood interactions as evidence that nutrient availability modifies the strength and direction of local biotic interactions.

#### Seedling survival model

To investigate how soil nutrients regulate neighborhood interactions during the seedling life stage, we fitted generalized linear mixed-effects models assuming a logit-link function and binomial error to examine the neighborhood effect on first-year survival for newly germinated seedlings during 2008 and 2023. Seedling neighborhood density was defined as the number of conspecific and heterospecific seedlings within the same quadrat. To examine whether neighborhood effects increase or decrease with soil nutrients, we included interaction terms between neighborhood variables and soil nutrients (SoilNut). The initial height of seedlings ($\text{IniHeigh}t_{\mathrm{jkt}}$) was included in the model as a covariate due to its strong influence on seedling survival.

To account for species-specific variation and spatiotemporal structure, we incorporated random effects for species identity ($\delta_{j}$), recruitment year ($\delta_{t}$), and 10 m–by–10 m subplot ($\delta_{k}$). These random effects absorb unmeasured variation shared within spatial units and years, thereby reducing spatiotemporal autocorrelation and mitigating potential confounding effects of spatially structured variation (e.g., elevation and slope) and temporally varying climate (e.g., temperature and precipitation). The seedling survival status ($\mathrm{su}r_{\mathrm{jkt}}$, survival or dead) for species k in quadrat j from recruitment year t follows a binomial distribution with probability $p_{\mathrm{jkt}}$

$$\mathrm{su}r_{\mathrm{jkt}}=\text{binomial}\left(p_{\mathrm{jkt}}\right)$$ (3)

We fitted the following model for each soil nutrient, respectively

$$\begin{array}{cc} \text{logit}\left(p_{\mathrm{jkt}}\right)= & \left(\text{Intercept}\right)+\mathrm{CS}D_{\mathrm{jkt}}+\mathrm{HS}D_{\mathrm{jkt}}+\text{SoilNu}t_{j}+\mathrm{CS}D_{\mathrm{jkt}}\times \\ & {\text{SoilNut}}_{j}+\mathrm{HS}D_{\mathrm{jkt}}\times {\text{SoilNut}}_{j}+\text{IniHeigh}t_{\mathrm{jkt}}+\delta_{j}+\delta_{k}+\delta_{t} \end{array}$$ (4)

where $\mathrm{CS}D_{\mathrm{jkt}}$ and $\mathrm{HS}D_{\mathrm{jkt}}$ represent the conspecific and heterospecific seedling neighborhood effects, respectively. $\text{SoilNu}t_{j}$ refers to the soil nutrient concentration predicted from kriging interpolation in quadrat j.

#### Coefficient interpretation

In line with the estimands defined above, coefficients of $\mathrm{CS}D_{\mathrm{jkt}}$ and $\mathrm{HS}D_{\mathrm{jkt}}$ quantify the magnitude and direction of neighborhood effects conditional on nutrient context, with negative coefficients indicating stronger negative density dependence (CNDD or HNDD). The interaction terms ($\mathrm{CS}D_{\mathrm{jkt}}\times {\text{SoilNut}}_{j}$) and ($\mathrm{HS}D_{\mathrm{jkt}}\times {\text{SoilNut}}_{j}$) quantify nutrient-dependent modification of neighborhood effects (i.e., how the strength/direction of density dependence changes along nutrient gradients). A significant positive interaction term indicates that CNDD or HNDD becomes weaker with increased nutrient availability (strengthened facilitation), and a significant negative interaction term suggests that higher nutrient availability strengthens CNDD or HNDD (strengthened competition). The coefficient of $\text{SoilNu}t_{j}$ represents an effect conditional on neighborhood context and is not interpreted as the total causal effect of nutrients on survival because the total effect may also operate indirectly through nutrient-driven changes in neighborhood structure.

#### Adult growth model

Similarly, adult tree growth was modeled as a function of neighborhood variables, soil nutrients, and their interactions, with initial DBH ($\text{IniDB}H_{\mathrm{ijt}}$) included as a covariate. Adult tree growth was quantified as the increase in DBH between consecutive censuses. Neighbors were defined as all adult trees within a 10-m radius of the focal tree, as interactions are expected to weaken beyond this distance (*86*). Conspecific or heterospecific adult neighborhood density was calculated using the formula $\sum \frac{BA_{\text{neighbors}}}{\text{Dist }\times \;BA_{\text{focal}}}$ (*87*), where $BA_{\text{focal}}$ represents the basal area of neighboring trees and Dist is the spatial distance between the focal tree and its neighbors. This index assumes that larger and closer trees impose a greater competitive effect on smaller trees.

We included individual identity ($\delta_{i}$) as a random effect to account for variation arising from repeated measurements of the same individuals. $\delta_{j}$ and $\delta_{k}$ represent random effects for species identity and quadrat, respectively, while the year effect ($\delta_{t}$) was included as a fixed effect. The rationality for specifying these effects is analogous to that in the seedling neighborhoods model described above. To avoid edge effects, only surviving trees located at least 10 m from the boundaries of the 50-ha plot were included in the analysis. All neighborhood variables were log-transformed before analysis to capture potential nonlinear relationships. All soil nutrient and neighborhood variables were centered and scaled before analysis to facilitate better comparison of coefficients. Adult growth was defined as

$$\Delta \text{DB}H_{\mathrm{ijk}\left(t\right)}={\mathrm{DBH}}_{\mathrm{ijk}\left(t+1\right)}-\mathrm{DB}H_{\mathrm{ijk}\left(t\right)}$$ (5)

We fitted the following model for each soil nutrient, respectively

$$\begin{array}{c} \Delta \text{DB}H_{\mathrm{ijk}\left(t\right)}=\left(\text{Intercept}\right)+\mathrm{CA}D_{\text{ijkt}}+\mathrm{HA}D_{\text{ijkt}}+\text{SoilNu}t_{j}+\mathrm{CA}D_{\text{ijkt}}\times \\ {\text{SoilNut}}_{j}+\mathrm{HA}D_{\text{ijkt}}\times {\text{SoilNut}}_{j}+\text{IniDB}H_{\mathrm{ijt}}+\delta_{i}+\delta_{j}+\delta_{k}+\delta_{t} \end{array}$$ (6)

#### Coefficient interpretation

As in the seedling models, $\mathrm{CA}D_{\text{ijkt}}$ and $\mathrm{HA}D_{\text{ijkt}}$ quantify the magnitude and direction of neighborhood effects conditional on nutrient level. The regression coefficients are interpreted similarly to those in the seedling-stage neighborhood models.

In summary, by specifying a DAG before modeling and aligning model structure and coefficient interpretation with that causal framework, our neighborhood analyses help us clarify how soil nutrients regulate interspecific and intraspecific interactions across life stages, offering insights into the mechanisms through which soil nutrients influence diversity and stability.

## Supplementary Materials

### This PDF file includes:

Supplementary Text Figs. S1 to S10 Tables S1 to S9 References
