# Supplementary material for: Soil phosphorus availability drives decade-scale stability across life stages in a subtropical forest
Source: Sci Adv. 2026 Apr 24;12(17):eaec5351. doi: 10.1126/sciadv.aec5351 (PMC13108531; doi:10.1126/sciadv.aec5351)
Supplement: Supplementary file 1 — Supplementary Text Figs. S1 to S10 Tables S1 to S9 References [file sciadv.aec5351_sm.pdf]

Science Advances
AAAS

Supplementary Materials for

Soil phosphorus availability drives decade-scale stability across life stages in a subtropical forest

Ganxin Feng *et al.*

Corresponding author: Minxia Liang, liangmx3@mail.sysu.edu.cn

Sci. Adv. **12**, eaec5351 (2026)
DOI: 10.1126/sciadv.aec5351

This PDF file includes:

Supplementary Text
Figs. S1 to S10
Tables S1 to S9
References

Supplementary Text

Text S1. Kriging interpolation

To generate the spatial prediction of soil N and P concentration across all seedling and adult quadrats, we applied universal kriging based on extensive field sampling and geostatistical modeling. In total, 1288 soil samples were collected from a 50-ha forest plot, including 588 regular samples (one from every alternate 20 m × 20 m quadrat) and 700 cluster samples (seven per cluster) designed to capture fine-scale spatial variation and anisotropy across the plot (see Fig S7). The kriging procedure for the 50-ha plot followed the same framework as in our previous study (*9*), where the interpolation methods for soil nutrients and their application to seedling quadrats were previously introduced.

Prior to interpolation, nutrient data were Box-Cox transformed to improve normality. Empirical semi-variograms were computed and fitted with appropriate theoretical models (e.g., spherical, exponential, and Gaussian) to capture spatial structure. The best-fitting model for each nutrient was selected based on the overall fit to the empirical variogram. Kriging was then performed over a regular prediction grid, incorporating spatial trends and accounting for spatial autocorrelation. This approach enabled continuous quadrat predictions of soil nutrient availability across the study area.

Text S2. Allometry growth equation

We estimated aboveground biomass based on DBH by using diameter-class specific allometry growth equation of subtropical evergreen broad-leaved forests (*88*). Aboveground biomass (AGB) per tree was calculated as

$$\text{AGB}=\begin{cases} 0.0500\times\text{DBH}^{2.5669},\ 0 < \text{DBH} \le 5\text{ cm} \\ 0.0513\times\text{DBH}^{2.6294},\ 5\text{ cm} < \text{DBH} \le 10\text{ cm} \\ 0.1138\times\text{DBH}^{2.2976},\ 10\text{ cm} < \text{DBH} \le 20\text{ cm} \\ 0.7007\times\text{DBH}^{1.7172},\ \text{DBH} > 20\text{ cm} \end{cases}$$

where DBH denotes the diameter at breast height.

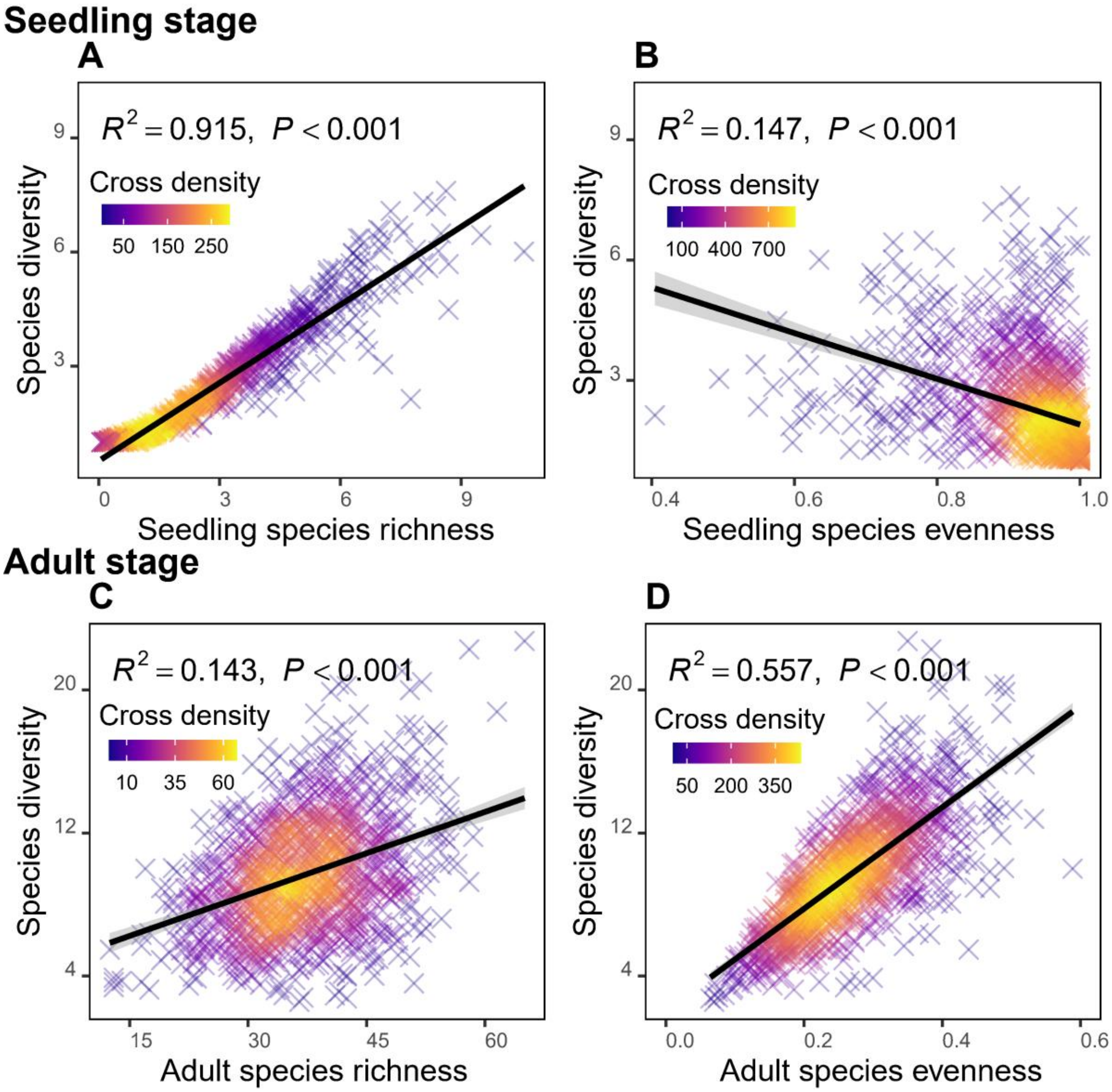

Fig. S1. Relationship between species richness, species evenness, and species diversity separately for seedling stage (A, B) and adult stage (C, D). Panel **A** and **B** show the relationship between species richness, species evenness and species diversity at seedling stage. Panel **C** and **D** show the relationship between species richness, species evenness and species diversity at adult stage. The solid lines represent significant relationships fitted by bivariate linear regression and the shades are the corresponding 95% confidence interval. Each cross represents one sampling unit: a 1 m × 1 m seedling quadrat across the six 1-ha seedling plot (seedling stage) or a 20 m × 20 m quadrat within the 50-ha plot (adult stage). Crosses are colored by cross density (upper-left color legend), with warmer colors indicating higher cross densities.

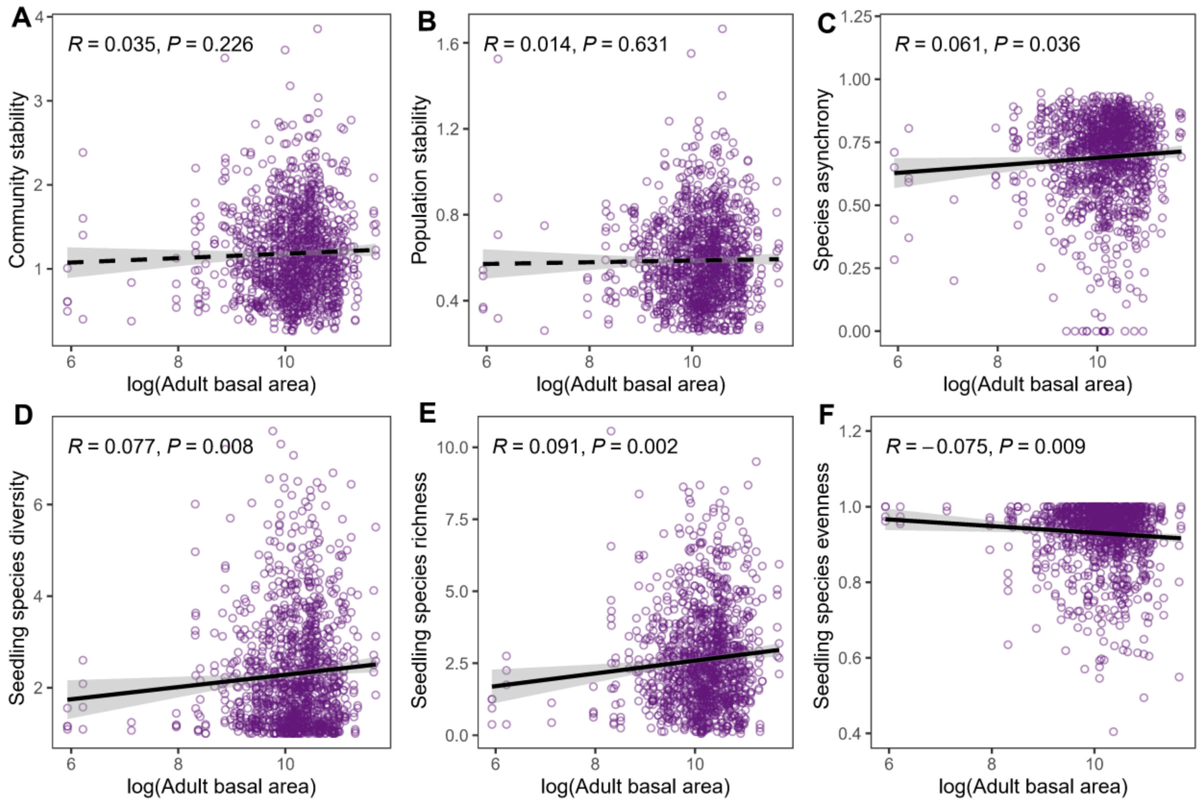

Fig. S2. Associations between an overstory structure proxy (adult basal area) and seedling diversity and stability metrics. Adult basal area was calculated within each 10 m × 10 m seedling subplot and log-transformed. Panels show relationships between log-transformed adult basal area and community stability (**A**), population stability (**B**), species asynchrony (**C**), species diversity (**D**), species richness (**E**), species evenness (**F**). Points represent 1 m × 1 m seedling quadrat. Lines show fitted relationships from separate linear models for each response; solid lines indicate statistically significant slopes ($P < 0.05$), whereas dashed lines indicate non-significant slopes ($P \geq 0.05$).

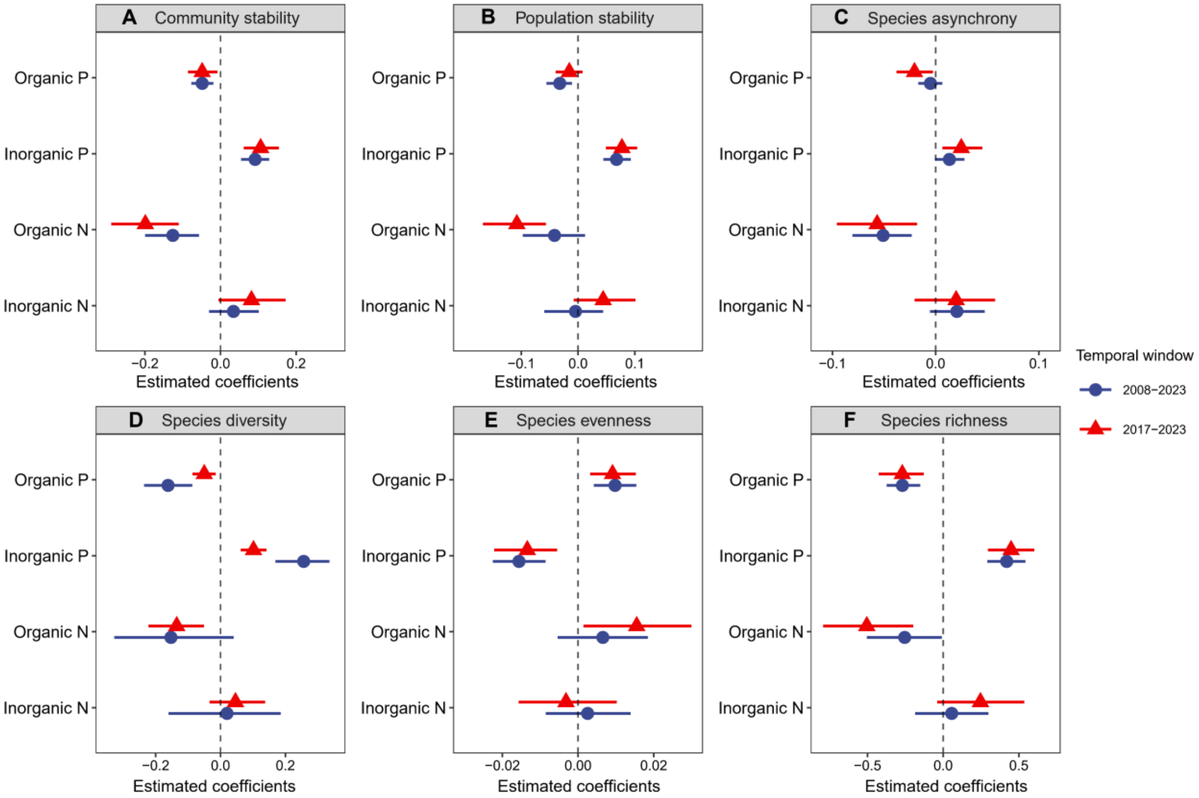

Fig. S3. Temporal alignment sensitivity for seedlings comparing regression coefficients between the full-period and aligned-window analyses. We recomputed seedling diversity and stability metrics within a temporally aligned window (2017–2023) matching the soil survey and refitted the same regression models used in the main analysis. Panels show results for community stability (**A**), population stability (**B**), species asynchrony (**C**), species diversity (**D**), species evenness (**E**), and species richness (**F**). In each panel, standardized regression coefficients estimated from the full-period analysis (2008–2023) are compared with those from the aligned-window analysis (2017–2023) for the main nutrient predictors. Coefficient directions were consistent between analysis windows, with expected changes in coefficient magnitude and uncertainty under the shorter aligned window.

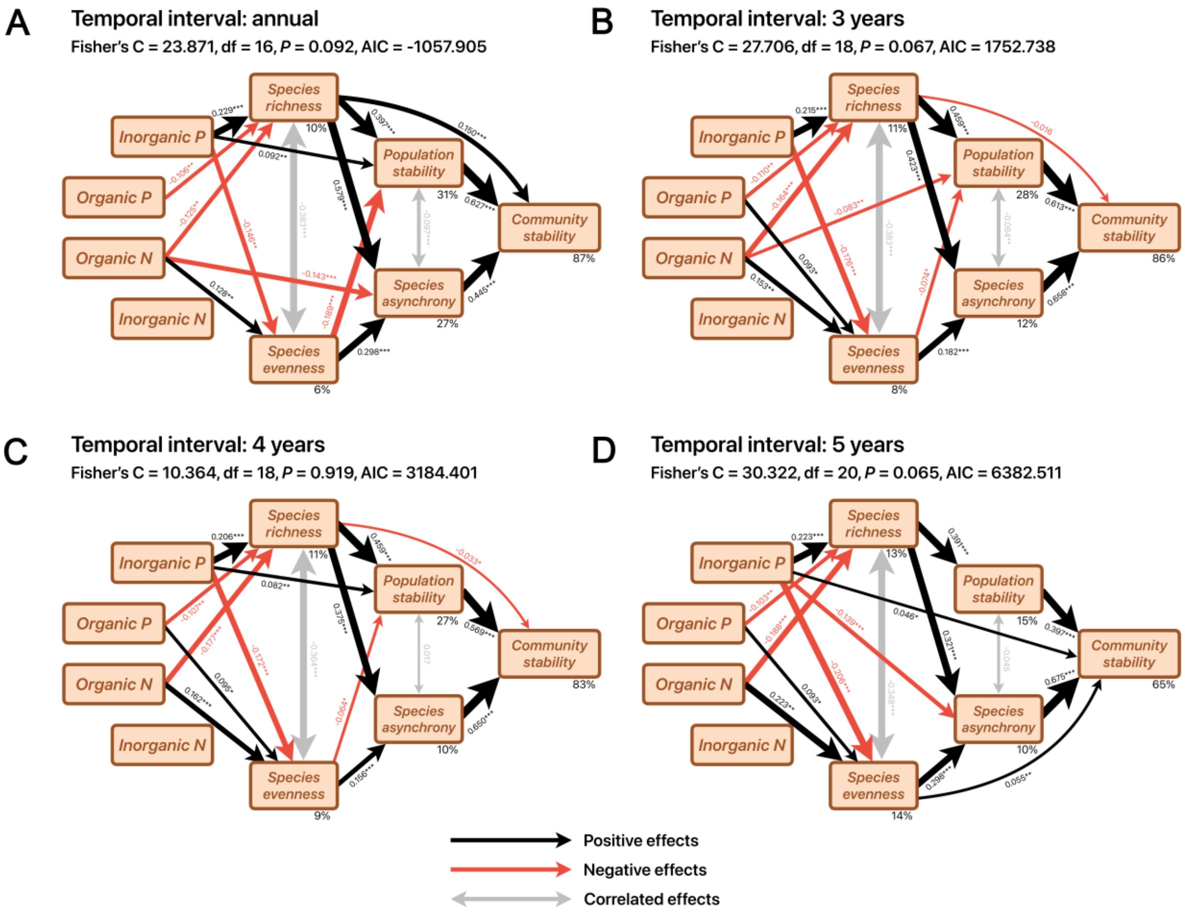

Fig. S4. Temporal-interval sensitivity analysis for seedlings showing robust pSEM pathways across census intervals. pSEM were refitted for seedling quadrats using community productivity and derived stability metrics calculated at four temporal resolutions: annual (**A**), and non-overlapping 3 years (**B**), 4 years (**C**), and 5 years (**D**) intervals. Standardized path coefficients are shown on arrows. Black and red arrows indicate positive and negative effects, respectively (* $P < 0.05$, ** $P < 0.01$, *** $P < 0.001$). Grey arrows indicate correlated paths. Numbers next to response variables indicate the proportion of variance explained (R^2). Model fit statistics (Fisher's C, degrees of freedom, P-value, and AIC) are reported above each panel.

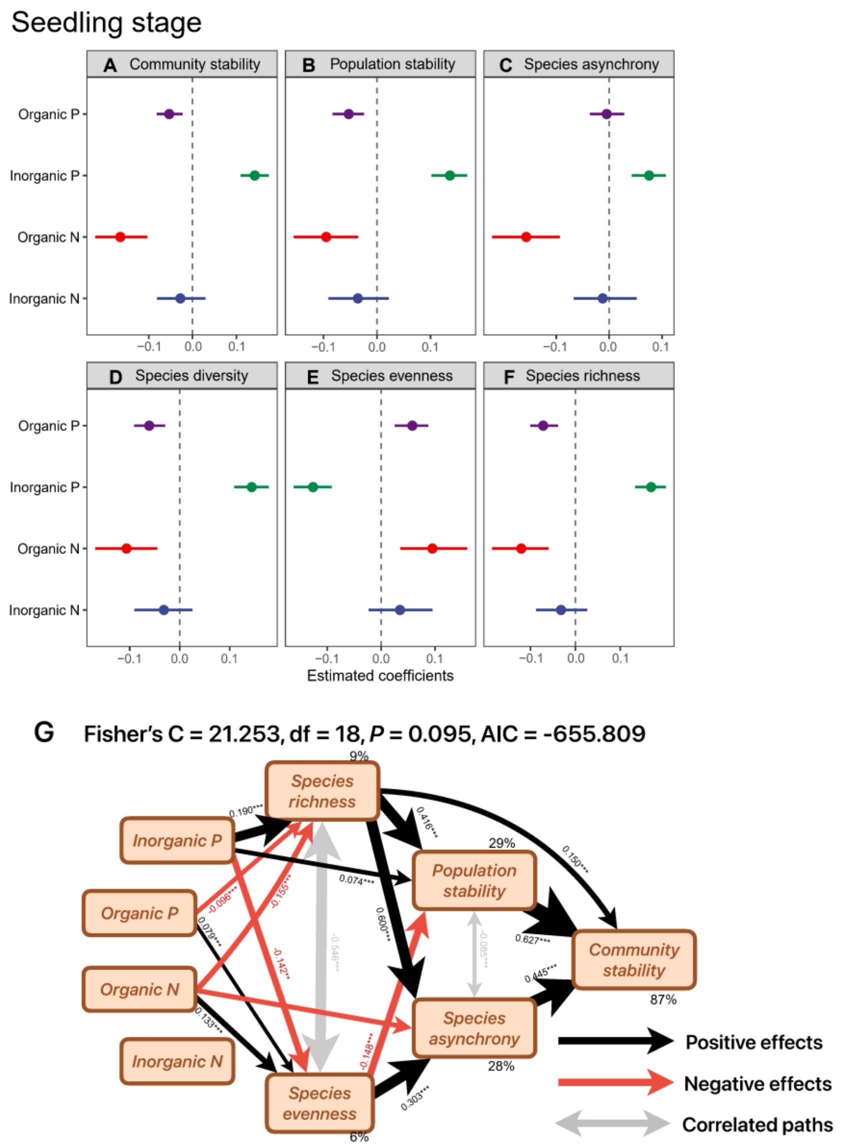

Fig. S5. Kriging uncertainty propagation for the seedling stage showing robust regression coefficients and pSEM pathways. We propagated kriging prediction uncertainty for soil nutrient forms into downstream analyses via Monte Carlo sampling and summarized results for the seedling stage. Panels **A**–**F** show standardized regression coefficients (points) and uncertainty intervals (horizontal bars show the 2.5th–97.5th percentile intervals) for the effects of nutrient forms on seedling community stability (**A**), population stability (**B**), species asynchrony (**C**), species diversity (**D**), species evenness (**E**), and species richness (**F**). Panel **G** shows the corresponding pSEM fitted using the same Monte Carlo–propagated nutrient predictors. In Panel **G**, arrow colors denote positive (black) and negative (red) effects and grey arrows indicate correlated paths; numbers on arrows are standardized path coefficients with significance denoted by asterisks (*$P < 0.05$, **$P < 0.01$, ***$P < 0.001$), and percentages next to response variables indicate explained variance (R^2). Fisher's C, degrees of freedom, *P*-value, and AIC are reported above the pSEM. Overall, incorporating kriging uncertainty did not alter the direction or qualitative interpretation of key nutrient-related effects relative to the main analysis.

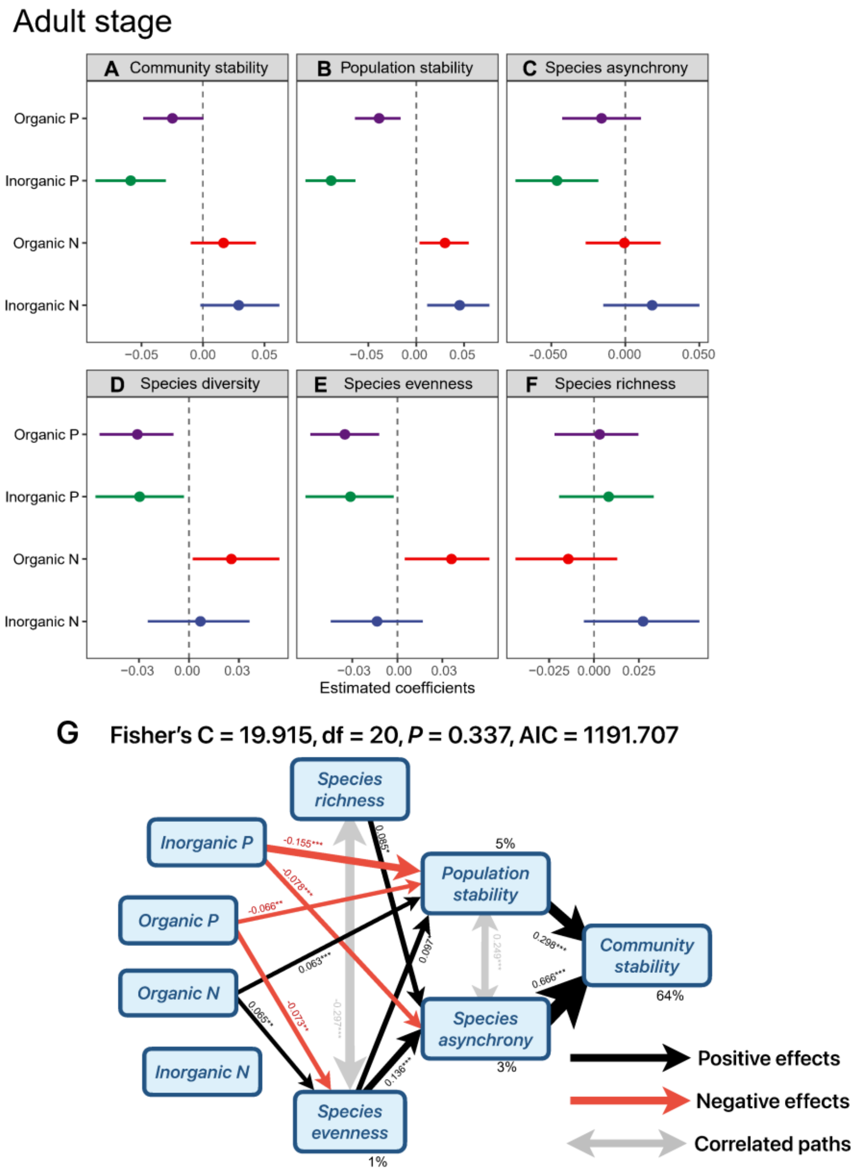

Fig. S6. Kriging uncertainty propagation for the adult stage showing robust regression coefficients and pSEM pathways. We propagated kriging prediction uncertainty for soil nutrient forms into downstream analyses via Monte Carlo sampling and summarized results for the adult stage. Panels **A**–**F** show standardized regression coefficients (points) and uncertainty intervals (horizontal bars show the 2.5th–97.5th percentile intervals) for the effects of nutrient forms on adult community stability (**A**), population stability (**B**), species asynchrony (**C**), species diversity (**D**), species evenness (**E**), and species richness (**F**). Panel **G** shows the corresponding pSEM fitted using the same Monte Carlo–propagated nutrient predictors. In Panel **G**, arrow colors denote positive (black) and negative (red) effects and grey arrows indicate correlated paths; numbers on arrows are standardized path coefficients with significance denoted by asterisks (**P* < 0.05, ***P* < 0.01, ****P* < 0.001), and percentages next to response variables indicate explained variance (R^2). Fisher's C, degrees of freedom, *P*-value, and AIC are reported above the pSEM. Overall, incorporating kriging uncertainty did not alter the direction or qualitative interpretation of key nutrient-related effects relative to the main analysis.

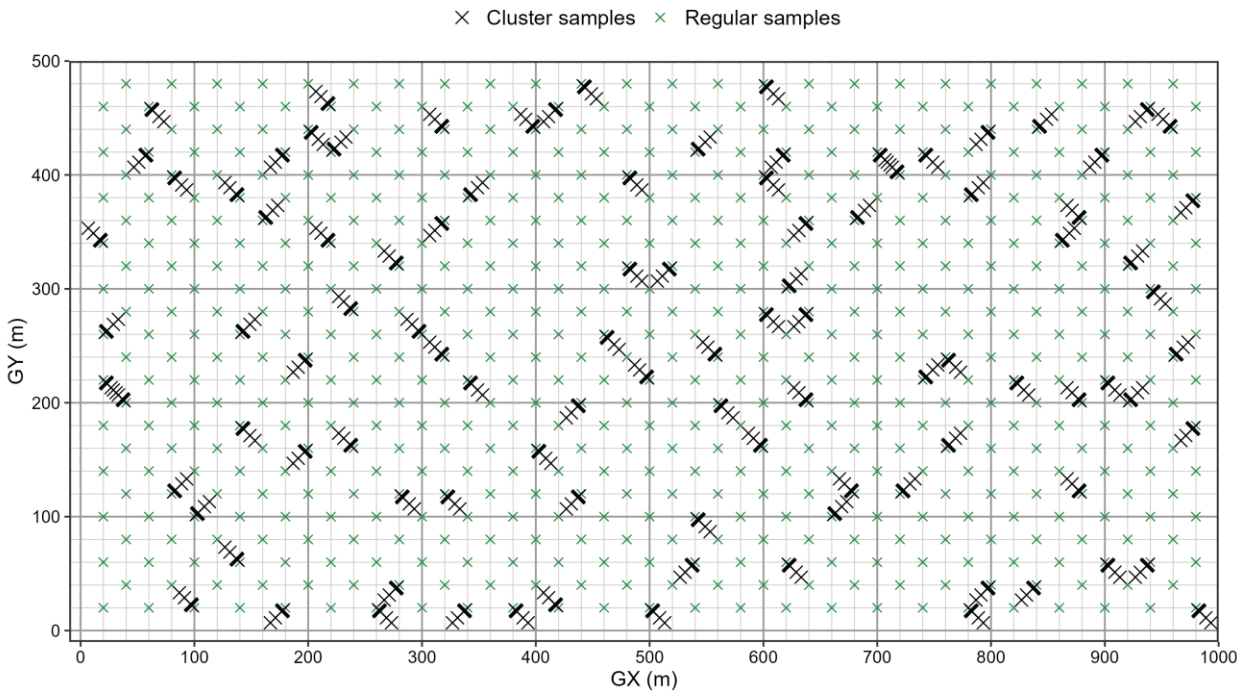

Fig. S7. Sampling design for soils on the 50-ha permanent plot sampling locations. Black crosses indicate regular soil samples, while green crosses denote cluster soil samples. In 2023, we collected 1288 soil samples within the 50-ha plot. Regular soil samples (n = 588) were obtained by collecting one sample from every alternate 20 m × 20 m quadrat. To capture directional variation in soil properties, we randomly selected two regular sampling points within each 100 m × 100 m plot. At each selected point, we collected seven additional soil samples at distances of 0.16 m, 0.33 m, 0.61 m, 1.03 m, 2.66 m, 6.22 m, and 7.67 m in a randomly chosen direction, resulting in 700 cluster soil samples.

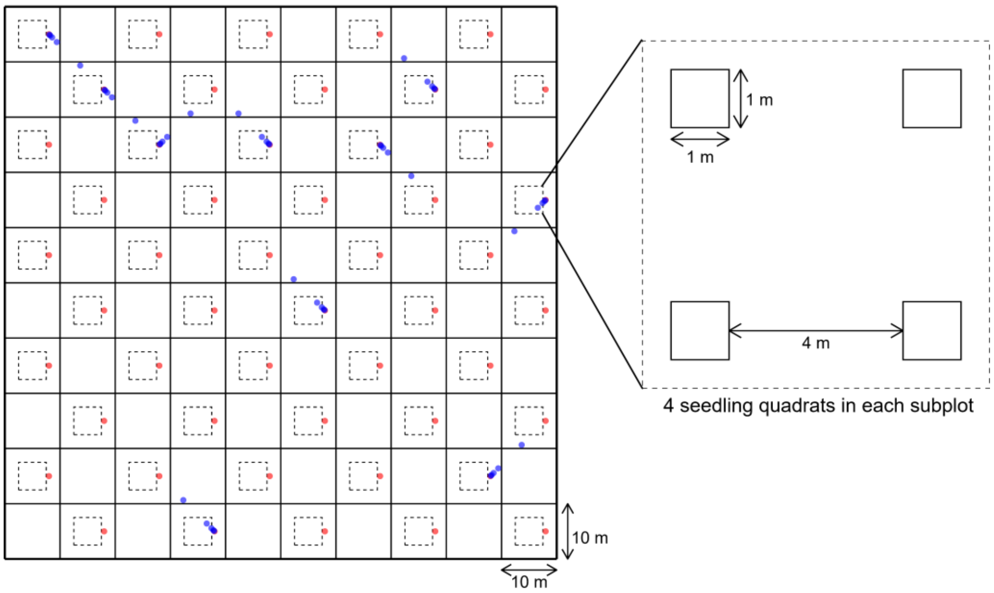

Fig. S8. Experiment design of 1-ha permanent plot and soil sampling locations, following the methodology of our previous study (*9*). Each 1-ha plot consists of fifty census stations, systematically arranged in alternating 10 m × 10 m subplots. Each station includes four 1 m × 1 m seedling quadrats. Soil samples were collected for each seedling quadrat, resulting in 50 regular samples (red points) per plot. To capture fine-scale spatial variation in soil properties, 20% of the sites (one out of every five sampling stations per column) were randomly selected for additional sampling. At these selected sites, five additional soil samples were taken at distances of 0.1 m, 0.3 m, 0.8 m, 2 m, and 8 m from the original location in a randomly chosen direction, resulting in 50 cluster soil samples (blue points).

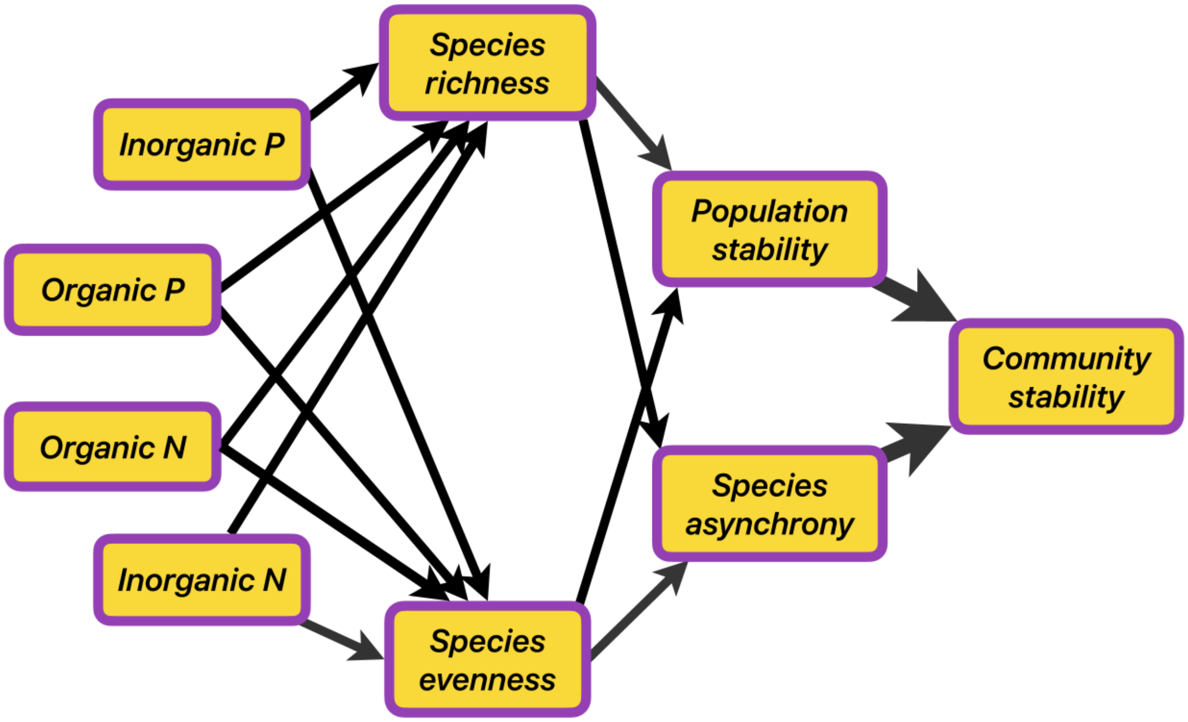

Fig. S9. Concept framework showing the effects of soil nutrients on diversity, asynchrony, and temporal stability. We hypothesize that soil nutrients influence community temporal stability and its two components—population stability and species asynchrony—by altering two key components of species diversity: richness and evenness. In addition, the diagram indicates a small number of ecologically plausible direct effects (e.g., nutrient forms → population stability/species asynchrony and richness → community stability) that are retained in the final pSEM where supported.

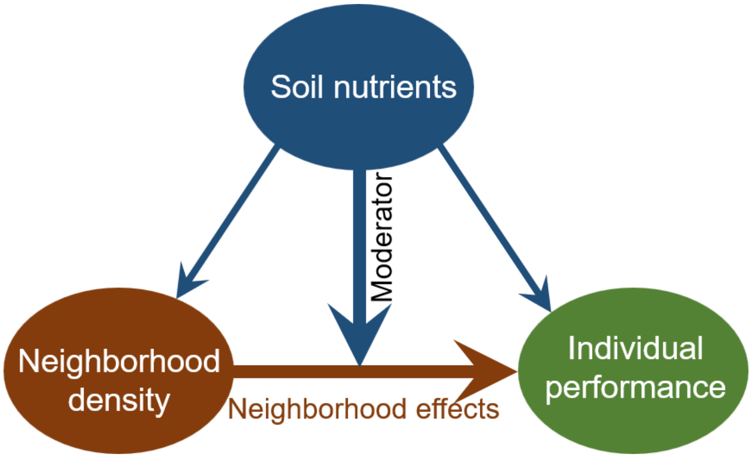

Fig. S10. Directed acyclic graph (DAG) of soil nutrient effects on neighborhood density and individual performance. This DAG summarizes the assumed causal relationships among soil nutrients, neighborhood density, and individual performance (seedling survival or adult growth). Soil nutrients may influence individual performance both directly and indirectly by shaping neighborhood density. Neighborhood density affects individual performance by capturing local biotic interactions (competition/facilitation). Soil nutrients are further hypothesized to modify the magnitude and direction of neighborhood effects (Moderator), which is tested in the neighborhood models via nutrient × neighborhood interaction terms. Under this framework, nutrient main effects in neighborhood models are interpreted as effects conditional on neighborhood context rather than as total nutrient effects.

Table S1. The statistical parameters of diversity-stability relationships across life stages. Species diversity was calculated as the exponential of Shannon entropy. Species evenness was measured by the ratio between species diversity and species richness with a range from 0 to 1. Significant values are highlighted in bold (*P* < 0.05).

Dependent variable	Life stage	Independent variable	Estimate	Std.Error	t value	Pr(>|z|)	R^2
Community stability	Seedling	Seedling species diversity	0.242	0.008	28.690	**<0.001**	0.408
		Seedling species richness	0.185	0.005	32.150	**<0.001**	0.464
		Seedling species evenness	-1.847	0.155	-11.920	**<0.001**	0.106
		Population stability	1.158	0.022	51.570	**<0.001**	0.690
		Species asynchrony	1.843	0.056	32.540	**<0.001**	0.470
	Adult	Adult species diversity	0.042	0.009	4.231	**<0.001**	0.014
		Adult species richness	0.002	0.004	0.649	0.516	0.000
		Adult species evenness	1.538	0.379	4.055	**<0.001**	0.013
		Population stability	1.437	0.074	19.277	**<0.001**	0.229
		Species asynchrony	2.940	0.074	39.731	**<0.001**	0.558
Population stability	Seedling	Seedling species diversity	0.136	0.006	20.000	**<0.001**	0.251
		Seedling species richness	0.111	0.004	23.910	**<0.001**	0.324
		Seedling species evenness	-1.595	0.108	-14.739	**<0.001**	0.154
	Adult	Adult species diversity	0.012	0.003	3.632	**<0.001**	0.010
		Adult species richness	-0.000	0.001	-0.129	0.898	0.000
		Adult species evenness	0.516	0.126	4.088	**<0.001**	0.013
Species asynchrony	Seedling	Seedling species diversity	0.064	0.003	17.660	**<0.001**	0.207
		Seedling species richness	0.046	0.002	17.700	**<0.001**	0.208
		Seedling species evenness	-0.146	0.060	-2.406	**0.016**	0.004
	Adult	Adult species diversity	0.012	0.002	5.048	**<0.001**	0.020
		Adult species richness	0.001	0.001	1.538	0.124	0.001
		Adult species evenness	0.394	0.096	4.091	**<0.001**	0.013

Table S2. Associations between community composition and community stability across life stages. PERMANOVA were performed on Bray–Curtis dissimilarities with 999 permutations. Mantel tests relate pairwise compositional dissimilarity (total β) and its decomposed components (balanced variation in abundance; abundance gradients) to pairwise differences in community stability using Spearman correlations (999 permutations).

**Life stage**	**Test**	**Component**	**Statistic**	R^2	***P* value**
Seedling stage	PERMANOVA	Total β diversity	*F* = 40.583	0.032	0.001***
	Mantel test	Total β diversity	ρ = 0.055	-	0.001***
	Mantel test	Balance variation	ρ = -0.026	-	1.000
	Mantel test	Abundance gradient	ρ = 0.088	-	0.001***
Adult stage	PERMANOVA	Total β diversity	*F* = 7.601	0.006	0.001***
	Mantel test	Total β diversity	ρ = 0.017	-	0.061
	Mantel test	Balance variation	ρ = 0.009	-	0.156
	Mantel test	Abundance gradient	ρ = 0.011	-	0.139

Table S3. The effects of different chemical forms of soil nutrients on diversity and stability across life stages. Species diversity was calculated as the exponential of Shannon entropy. Species evenness was measured by the ratio between species diversity and species richness ranging from 0 to 1. Significant values are highlighted in bold (*P* < 0.05).

Dependent variable	Life stage	Independent variable	Estimate	Std.Error	t value	Pr(>|z|)
Community stability	Seedling	Organic P	-0.049	0.015	-3.248	**0.001**
		Inorganic P	0.092	0.016	5.465	**<0.001**
		Organic N	-0.127	0.036	-3.542	**<0.001**
		Inorganic N	0.035	0.034	1.036	0.301
	Adult	Organic P	-0.133	0.040	-3.330	**<0.001**
		Inorganic P	-0.145	0.037	-3.921	**<0.001**
		Organic N	0.017	0.041	0.401	0.689
		Inorganic N	0.092	0.051	1.822	0.069
Population stability	Seedling	Organic P	-0.033	0.011	-2.953	**0.003**
		Inorganic P	0.069	0.012	5.596	**<0.001**
		Organic N	-0.040	0.026	-1.532	0.126
		Inorganic N	-0.006	0.025	-0.241	0.809
	Adult	Organic P	-0.072	0.013	-5.607	**<0.001**
		Inorganic P	-0.067	0.012	-5.638	**<0.001**
		Organic N	0.016	0.013	1.224	0.221
		Inorganic N	0.047	0.016	2.878	**0.004**
Species asynchrony	Seedling	Organic P	-0.005	0.006	-0.862	0.389
		Inorganic P	0.013	0.006	2.079	**0.038**
		Organic N	-0.052	0.014	-3.726	**<0.001**
		Inorganic N	0.021	0.013	1.552	0.121
	Adult	Organic P	-0.019	0.010	-1.880	0.060
		Inorganic P	-0.034	0.010	-3.520	**<0.001**
		Organic N	-0.014	0.011	-1.287	0.198
		Inorganic N	0.019	0.013	1.498	0.134
Species diversity	Seedling	Organic P	-0.160	0.041	-3.933	**<0.001**
		Inorganic P	0.257	0.045	5.682	**<0.001**

		Organic N	-0.149	0.097	-1.538	0.124
		Inorganic N	0.016	0.093	0.170	0.865
	Adult	Organic P	-0.494	0.113	-4.359	**<0.001**
		Inorganic P	-0.075	0.105	-0.709	0.478
		Organic N	0.314	0.117	2.684	**0.007**
		Inorganic N	0.093	0.143	0.650	0.516
Species evenness	Seedling	Organic P	0.010	0.003	3.509	**<0.001**
		Inorganic P	-0.015	0.003	-5.042	**<0.001**
		Organic N	0.007	0.007	1.013	0.311
		Inorganic N	0.002	0.006	0.389	0.697
	Adult	Organic P	-0.012	0.003	-4.024	**<0.001**
		Inorganic P	-0.003	0.003	-1.163	0.245
		Organic N	0.014	0.003	4.546	**<0.001**
		Inorganic N	-0.005	0.004	-1.377	0.169
Species richness	Seedling	Organic P	-0.265	0.056	-4.740	**<0.001**
		Inorganic P	0.421	0.062	6.768	**<0.001**
		Organic N	-0.253	0.133	-1.902	0.057
		Inorganic N	0.052	0.128	0.402	0.688
	Adult	Organic P	-0.295	0.281	-1.048	0.295
		Inorganic P	0.327	0.261	1.255	0.210
		Organic N	-0.809	0.290	-2.787	**0.005**
		Inorganic N	1.127	0.356	3.168	**0.002**

Table S4. Regression coefficients of seedling survival models with multiple neighborhood variables. Significant values are highlighted in bold ($P < 0.05$).

	Estimate	Std.Error	Z value	Pr(>|z|)
**Seedling neighborhood model for organic P**				
(Intercept)	0.355	0.195	1.823	**<0.001**
Organic P	-0.082	0.031	-2.640	**0.008**
Conspecific seedling density	-0.073	0.024	-2.979	**0.002**
Heterospecific seedling density	0.036	0.023	1.525	0.127
Conspecific seedling density × Organic P	0.017	0.023	0.776	0.437
Heterospecific seedling density × Organic P	-0.042	0.021	-1.990	**0.046**
IniHeight	1.374	0.065	20.846	**<0.001**
**Seedling neighborhood model for inorganic P**				
(Intercept)	0.330	0.197	1.673	0.094
Inorganic P	-0.122	0.037	-3.309	**<0.001**
Conspecific seedling density	-0.062	0.024	-2.506	**0.012**
Heterospecific seedling density	0.048	0.024	2.039	**0.041**
Conspecific seedling density × Inorganic P	-0.032	0.023	-1.407	0.159
Heterospecific seedling density × Inorganic P	0.057	0.022	2.534	**0.011**
IniHeight	1.376	0.065	20.887	**<0.001**
**Seedling neighborhood model for organic N**				
(Intercept)	0.328	0.195	1.678	0.093
Organic N	0.048	0.041	1.177	0.239
Conspecific seedling density	-0.066	0.024	-2.681	**0.007**
Heterospecific seedling density	0.039	0.024	1.658	0.097
Conspecific seedling density × Organic N	0.065	0.023	2.792	**0.005**
Heterospecific seedling density × Organic N	-0.112	0.023	-4.811	**<0.001**
IniHeight	1.379	0.066	20.885	**<0.001**
**Seedling neighborhood model for inorganic N**				
(Intercept)	0.330	0.195	1.693	0.090
Inorganic N	0.054	0.037	1.456	0.145
Conspecific seedling density	-0.069	0.024	-2.804	**0.005**
Heterospecific seedling density	0.040	0.024	1.670	0.094
Conspecific seedling density × Inorganic N	0.068	0.022	3.003	**0.002**
Heterospecific seedling density × Inorganic N	-0.104	0.023	-4.522	**<0.001**
IniHeight	1.379	0.066	20.885	**<0.001**

Table S5. Regression coefficients of adult growth models with multiple neighborhood variables. Significant variables are highlighted in bold ($P < 0.05$).

	Estimate	Std.Error	t value	Pr(>|z|)
**Adult neighborhood model for organic P**				
(Intercept)	0.086	0.002	33.461	**<0.001**
Organic P	0.004	0.000	5.415	**<0.001**
Conspecific adult density	-0.005	0.000	-20.042	**<0.001**
Heterospecific adult density	-0.025	0.000	-65.877	**<0.001**
Conspecific adult density × Organic P	0.000	0.000	0.131	0.896
Heterospecific adult density × Organic P	-0.001	0.000	-5.049	**<0.001**
IniDBH	-0.034	0.000	-81.828	**<0.001**
**Adult neighborhood model for inorganic P**				
(Intercept)	0.086	0.002	33.442	**<0.001**
Inorganic P	-0.007	0.000	-10.372	**<0.001**
Conspecific adult density	-0.006	0.000	-20.228	**<0.001**
Heterospecific adult density	-0.025	0.000	-66.423	**<0.001**
Conspecific adult density × Inorganic P	0.000	0.000	2.686	**0.007**
Heterospecific adult density × Inorganic P	0.005	0.000	24.852	**<0.001**
IniDBH	0.034	0.000	82.076	**<0.001**
**Adult neighborhood model for organic N**				
(Intercept)	0.085	0.002	33.170	**<0.001**
Organic N	0.005	0.000	7.483	**<0.001**
Conspecific adult density	-0.006	0.000	-20.551	**<0.001**
Heterospecific adult density	-0.025	0.000	-65.706	**<0.001**
Conspecific adult density × Organic N	-0.001	0.000	-4.177	**<0.001**
Heterospecific adult density × Organic N	-0.007	0.000	-31.427	**<0.001**
IniDBH	0.034	0.000	82.059	**<0.001**
**Adult neighborhood model for inorganic N**				
(Intercept)	0.086	0.002	33.342	**<0.001**
Inorganic N	0.007	0.000	10.377	**<0.001**
Conspecific adult density	-0.006	0.000	-20.406	**<0.001**
Heterospecific adult density	-0.025	0.000	-65.660	**<0.001**
Conspecific adult density × Inorganic N	-0.000	0.000	-2.863	**0.004**
Heterospecific adult density × Inorganic N	-0.006	0.000	-27.841	**<0.001**
IniDBH	0.034	0.000	82.450	**<0.001**

Table S6. Reverse-direction sensitivity analysis showing strong spatial dependence in soil nutrient forms and improved fit of SAR error models relative to OLS. Each nutrient form (response variable) was modeled as a function of adult tree diversity, productivity, stability-related variables (2011–2021) using (i) OLS and (ii) SAR error models accounting for spatial autocorrelation among 20 m ×20 m quadrats. Pseudo-R^2 is calculated as $\mathbf{1 - \sigma^2_{SAR} / \sigma^2_{OLS}}$, where $\boldsymbol{\sigma^2}$ is residual variance. ΔAIC = AIC(OLS) − AIC(SAR); larger values indicate stronger improvement when accounting for spatial structure. All variables were standardized (z-scored) prior to modeling.

	**Organic P**	**Inorganic P**	**Organic N**	**Inorganic N**
R^2 (OLS)	0.190	0.038	0.079	0.115
σ^2_{OLS}	0.810	0.962	0.921	0.885
λ (SAR)	0.990	0.977	0.989	0.998
σ^2_{SAR}	0.087	0.144	0.069	0.047
Pseudo-R^2	0.893	0.850	0.925	0.947
AIC (OLS)	3293.567	3507.952	3453.896	3403.628
AIC (SAR)	859.183	1463.733	571.546	116.195
ΔAIC	2434.384	2044.219	2882.350	3287.432

Table S7. Reverse-direction sensitivity analysis showing standardized effects of adult vegetation-related variables on nutrient forms after accounting for spatial autocorrelation using SAR error models. Entries are standardized regression coefficients (β) from SAR error models; *P*-values are based on asymptotic z-tests. Only fixed effects are shown; λ and model fit statistics are reported in Table S6. Predictors: species richness, evenness, species asynchrony, population stability, temporal mean productivity, and temporal SD (standard deviation) of productivity. Significant variables are highlighted in bold ($P < 0.05$).

Predictor	Response variable	Estimate	Std.Error	Z value	Pr(>|z|)
Adult species richness	Inorganic P	0.010	0.011	0.927	0.354
	Organic P	0.010	0.014	0.728	0.467
	Inorganic N	0.003	0.010	0.286	0.775
	Organic N	-0.012	0.008	-1.557	0.120
Adult species evenness	Inorganic P	-0.000	0.009	-0.033	0.973
	Organic P	-0.014	0.012	-1.206	0.228
	Inorganic N	0.016	0.008	2.016	0.044
	Organic N	0.016	0.007	2.439	0.015
Species asynchrony	Inorganic P	0.004	0.015	0.303	0.762
	Organic P	0.003	0.019	0.160	0.873
	Inorganic N	0.001	0.013	0.058	0.953
	Organic N	0.003	0.011	0.252	0.801
Population stability	Inorganic P	-0.001	0.012	-0.077	0.939
	Organic P	-0.002	0.016	-0.116	0.908
	Inorganic N	0.000	0.011	0.001	1.000
	Organic N	-0.004	0.009	-0.398	0.691
Temporal mean of productivity	Inorganic P	-0.044	0.015	-2.960	0.003
	Organic P	-0.063	0.019	-3.267	0.001
	Inorganic N	0.019	0.013	1.413	0.158
	Organic N	0.040	0.011	3.645	<0.001
Temporal SD of productivity	Inorganic P	0.017	0.018	0.960	0.337
	Organic P	0.037	0.023	1.612	0.107
	Inorganic N	-0.001	0.016	-0.082	0.934
	Organic N	-0.004	0.013	-0.268	0.789

Table S8. Beta regression (logit link) analyses for asynchrony and evenness (range from 0 to 1) across life stages: bivariate relationships with diversity metrics and multivariate nutrient models. Significant values are highlighted in bold ($P < 0.05$).

Dependent variable	Life stage	Independent variable	Estimate	Std.Error	Z value	Pr(>|z|)
Species asynchrony	Seedling	Seedling species diversity	0.330	0.020	16.156	**<0.001**
		Seedling species richness	0.252	0.014	17.548	**<0.001**
		Seedling species evenness	-1.092	0.308	-3.552	**<0.001**
	Adult	Adult species diversity	0.043	0.010	4.207	**<0.001**
		Adult species richness	0.005	0.004	1.141	0.254
		Adult species evenness	1.394	0.390	3.576	**<0.001**
Species asynchrony	Seedling	Organic P	0.008	0.029	0.268	0.788
		Inorganic P	0.063	0.033	1.920	0.055
		Organic N	-0.202	0.069	-2.907	**0.004**
		Inorganic N	0.057	0.067	0.858	0.391
	Adult	Organic P	-0.095	0.042	-2.272	**0.023**
		Inorganic P	-0.107	0.039	-2.785	**0.005**
		Organic N	-0.020	0.043	-0.465	0.642
		Inorganic N	0.070	0.053	1.339	0.181
Species evenness	Seedling	Organic P	0.056	0.031	1.812	0.070
		Inorganic P	-0.144	0.034	-4.267	**<0.001**
		Organic N	0.095	0.073	1.295	0.195
		Inorganic N	0.097	0.071	1.369	0.171
	Adult	Organic P	-0.062	0.015	-4.066	**<0.001**
		Inorganic P	-0.021	0.014	-1.504	0.133
		Organic N	0.070	0.015	4.548	**<0.001**
		Inorganic N	-0.028	0.019	-1.447	0.148

Table S9. Variance inflation factors (VIFs) for assessing multicollinearity in the pSEM component models. To evaluate potential multicollinearity among predictors, we calculated VIFs for every component model included in the pSEM, separately for seedling and adult quadrats. For seedling-stage mixed-effects submodels, VIFs were computed from the fixed-effect design matrix; for adult-stage spatial error submodels, VIFs were computed from the corresponding non-spatial design matrix with the same predictors. "VIF range" denotes the minimum–maximum VIF across predictors in a given component model, and "Mean VIF" is calculated across predictors within that component model.

Life stage	Component model	VIF range	Mean VIF
Seedling stage	Community stability ~ Population stability + Species asynchrony + Species richness	1.26-1.67	1.43
	Population stability ~ Species richness + Species evenness + Inorganic P + Organic P	1.13-1.41	1.27
	Species asynchrony ~ Species richness + Species evenness + Organic N	1.07~1.44	1.30
	Species richness ~ Inorganic P + Organic P + Organic N	1.22-1.62	1.44
	Species evenness ~ Inorganic P + Organic N	1.33-1.33	1.33
Adult stage	Community stability ~ Population stability + Species asynchrony	1.08-1.08	1.08
	Population stability ~ Inorganic P + Organic P + Organic N	1.13-1.24	1.20
	Species asynchrony ~ Species richness + Species evenness + Inorganic P	1.01-1.11	1.07
	Species evenness ~ Organic P + Organic N	1.05-1.05	1.05

REFERENCES

1. I. Donohue, H. Hillebrand, J. M. Montoya, O. L. Petchey, S. L. Pimm, M. S. Fowler, K. Healy, A. L. Jackson, M. Lurgi, D. McClean, N. E. O'Connor, E. J. O'Gorman, Q. Yang, Navigating the complexity of ecological stability. *Ecol. Lett.* **19**, 1172–1185 (2016).

2. C. M. Clark, D. Tilman, Loss of plant species after chronic low-level nitrogen deposition to prairie grasslands. *Nature* **451**, 712–715 (2008).

3. Y. Hautier, D. Tilman, F. Isbell, E. W. Seabloom, E. T. Borer, P. B. Reich, Anthropogenic environmental changes affect ecosystem stability via biodiversity. *Science* **348**, 336–340 (2015).

4. F. I. Isbell, H. W. Polley, B. J. Wilsey, Biodiversity, productivity and the temporal stability of productivity: Patterns and processes. *Ecol. Lett.* **12**, 443–451 (2009).

5. M. Loreau, C. de Mazancourt, Biodiversity and ecosystem stability: A synthesis of underlying mechanisms. *Ecol. Lett.* **16**, 106–115 (2013).

6. P. A. Fay, S. M. Prober, W. S. Harpole, J. M. Knops, J. D. Bakker, E. T. Borer, E. M. Lind, A. S. MacDougall, E. W. Seabloom, P. D. Wragg, P. B. Adler, D. M. Blumenthal, Y. M. Buckley, C. Chu, E. E. Cleland, S. L. Collins, K. F. Davies, G. Du, X. Feng, J. Firn, D. S. Gruner, N. Hagenah, Y. Hautier, R. W. Heckman, V. L. Jin, K. P. Kirkman, J. Klein, L. M. Ladwig, Q. Li, R. L. McCulley, B. A. Melbourne, C. E. Mitchell, J. L. Moore, J. W. Morgan, A. C. Risch, M. Schutz, C. J. Stevens, D. A. Wedin, L. H. Yang, Grassland productivity limited by multiple nutrients. *Nat. Plants* **1**, 15080 (2015).

7. W. S. Harpole, L. L. Sullivan, E. M. Lind, J. Firn, P. B. Adler, E. T. Borer, J. Chase, P. A. Fay, Y. Hautier, H. Hillebrand, A. S. MacDougall, E. W. Seabloom, R. Williams, J. D. Bakker, M. W. Cadotte, E. J. Chaneton, C. Chu, E. E. Cleland, C. D'Antonio, K. F. Davies, D. S. Gruner, N. Hagenah, K. Kirkman, J. M. Knops, K. J. La Pierre, R. L. McCulley, J. L. Moore, J. W. Morgan, S. M. Prober, A. C. Risch, M. Schuetz, C. J. Stevens, P. D. Wragg, Addition of multiple limiting resources reduces grassland diversity. *Nature* **537**, 93–96 (2016).

8. Z. Xu, L. Jiang, H. Ren, X. Han, Opposing responses of temporal stability of aboveground and belowground net primary productivity to water and nitrogen enrichment in a temperate grassland. *Glob. Chang. Biol.* **30**, e17071 (2024).

9. M. Liang, Y. Zheng, D. Johnson, D. F. R. P. Burslem, L. Shi, J. Zhang, S. Yu, X. Liu, Long-term stability of sapling dynamics is regulated by soil phosphorus availability in subtropical forest. *J. Ecol.* **112**, 673–686 (2024).

10. Q. Chen, S. Wang, E. W. Seabloom, F. Isbell, E. T. Borer, J. D. Bakker, S. Bharath, C. Roscher, P. L. Peri, S. A. Power, I. Donohue, C. Stevens, A. Ebeling, C. Nogueira, M. C. Caldeira, A. S. MacDougall, J. L. Moore, S. Bagchi, A. Jentsch, M. Tedder, K. Kirkman, J. Alberti, Y. Hautier, Change in functional trait diversity mediates the effects of nutrient addition on grassland stability. *J. Ecol.* **112**, 2598–2612 (2024).

11. Q. Chen, S. Wang, E. W. Seabloom, A. S. MacDougall, E. T. Borer, J. D. Bakker, I. Donohue, J. M. H. Knops, J. W. Morgan, O. Carroll, M. Crawley, M. N. Bugalho, S. A. Power, A. Eskelinen, R. Virtanen, A. C. Risch, M. Schutz, C. Stevens, M. C. Caldeira, S. Bagchi, J. Alberti, Y. Hautier, Nutrients and herbivores impact grassland stability across spatial scales through different pathways. *Glob. Change Biol.* **28**, 2678–2688 (2022).

12. Y. Hautier, P. Zhang, M. Loreau, K. R. Wilcox, E. W. Seabloom, E. T. Borer, J. E. K. Byrnes, S. E. Koerner, K. J. Komatsu, J. S. Lefcheck, A. Hector, P. B. Adler, J. Alberti, C. A. Arnillas, J. D. Bakker, L. A. Brudvig, M. N. Bugalho, M. Cadotte, M. C. Caldeira, O. Carroll, M. Crawley, S. L. Collins, P. Daleo, L. E. Dee, N. Eisenhauer, A. Eskelinen, P. A. Fay, B. Gilbert, A. Hansar, F. Isbell, J. M. H. Knops, A. S. MacDougall, R. L. McCulley, J. L. Moore, J. W. Morgan, A. S. Mori, P. L. Peri, E. T. Pos, S. A. Power, J. N. Price, P. B. Reich, A. C. Risch, C. Roscher, M. Sankaran, M. Schutz, M. Smith, C. Stevens, P. M. Tognetti, R. Virtanen, G. M. Wardle, P. A. Wilfahrt, S. Wang, General destabilizing effects of eutrophication on grassland productivity at multiple spatial scales. *Nat. Commun.* **11**, 5375 (2020).

13. Y. Zhang, J. Feng, M. Loreau, N. He, X. Han, L. Jiang, Nitrogen addition does not reduce the role of spatial asynchrony in stabilising grassland communities. *Ecol. Lett.* **22**, 563–571 (2019).

14. Y. Zhang, M. Loreau, X. Lu, N. He, G. Zhang, X. Han, Nitrogen enrichment weakens ecosystem stability through decreased species asynchrony and population stability in a temperate grassland. *Glob. Change Biol.* **22**, 1445–1455 (2016).

15. Y. Wang, C. Wang, F. Ren, X. Jing, W. Ma, J. S. He, L. Jiang, Asymmetric response of aboveground and belowground temporal stability to nitrogen and phosphorus addition in a Tibetan alpine grassland. *Glob. Change Biol.* **29**, 7072–7084 (2023).

16. X. Qiao, T. Lamy, S. Wang, Y. Hautier, Y. Geng, H. J. White, N. Zhang, Z. Zhang, C. Zhang, X. Zhao, K. von Gadow, Latitudinal patterns of forest ecosystem stability across spatial scales as affected by biodiversity and environmental heterogeneity. *Glob. Change Biol.* **29**, 2242–2255 (2023).

17. F. Schnabel, X. Liu, M. Kunz, K. E. Barry, F. J. Bongers, H. Bruelheide, A. Fichtner, W. Hardtle, S. Li, C. T. Pfaff, B. Schmid, J. A. Schwarz, Z. Tang, B. Yang, J. Bauhus, G. von Oheimb, K. Ma, C. Wirth, Species richness stabilizes productivity via asynchrony and drought-tolerance diversity in a large-scale tree biodiversity experiment. *Sci. Adv.* **7**, eabk1643 (2021).

18. X. Qiao, Y. Geng, C. Zhang, Z. Han, Z. Zhang, X. Zhao, K. von Gadow, Spatial asynchrony matters more than alpha stability in stabilizing ecosystem productivity in a large temperate forest region. *Glob. Ecol. Biogeogr.* **31**, 1133–1146 (2022).

19. J. H. Connell, P. T. Green, Seedling dynamics over thirty-two years in a tropical rain forest tree. *Ecology* **81**, 568–584 (2000).

20. A. Favero, A. Daigneault, B. Sohngen, Forests: Carbon sequestration, biomass energy, or both? *Sci. Adv.* **6**, eaay6792 (2020).

21. D. Johnson, X. Liu, D. Burslem, Symbiotic control of canopy dominance in subtropical and tropical forests. *Trends Plant Sci.* **28**, 995–1003 (2023).

22. J. Ren, S. Fang, Q. W. Wang, H. Liu, F. Lin, J. Ye, Z. Hao, X. Wang, C. Fortunel, Ontogeny influences tree growth response to soil fertility and neighbourhood crowding in an old-growth temperate forest. *Ann. Bot.* **131**, 1061–1072 (2023).

23. B. Liu, C. Zhang, J. Deng, B. Zhang, F. Chen, W. Chen, X. Fang, J. Li, K. Zu, W. Bu, Response of tree growth to nutrient addition is size dependent in a subtropical forest. *Sci. Total Environ.* **923**, 171501 (2024).

24. D. A. King, "Size-related changes in tree proportions and their potential influence on the course of height growth" in *Size- and Age-Related Changes in Tree Structure and Function*, F. C. Meinzer, B. Lachenbruch, T. E. Dawson, Eds. (Springer, 2011), pp. 165–191.

25. D. Tilman, P. B. Reich, J. M. Knops, Biodiversity and ecosystem stability in a decade-long grassland experiment. *Nature* **441**, 629–632 (2006).

26. Q. Xu, X. Yang, Y. Yan, S. Wang, M. Loreau, L. Jiang, Consistently positive effect of species diversity on ecosystem, but not population, temporal stability. *Ecol. Lett.* **24**, 2256–2266 (2021).

27. L. M. Thibaut, S. R. Connolly, Understanding diversity-stability relationships: Towards a unified model of portfolio effects. *Ecol. Lett.* **16**, 140–150 (2013).

28. S. Wang, P. Hong, P. B. Adler, E. Allan, Y. Hautier, B. Schmid, J. W. Spaak, Y. Feng, Towards mechanistic integration of the causes and consequences of biodiversity. *Trends Ecol. Evol.* **39**, 689–700 (2024).

29. X. Morin, L. Fahse, C. de Mazancourt, M. Scherer-Lorenzen, H. Bugmann, Temporal stability in forest productivity increases with tree diversity due to asynchrony in species dynamics. *Ecol. Lett.* **17**, 1526–1535 (2014).

30. F. Schnabel, J. A. Schwarz, A. Danescu, A. Fichtner, C. A. Nock, J. Bauhus, C. Potvin, Drivers of productivity and its temporal stability in a tropical tree diversity experiment. *Glob. Change Biol.* **25**, 4257–4272 (2019).

31. M. O. Hill, Diversity and evenness: A unifying notation and its consequences. *Ecology* **54**, 427–432 (1973).

32. H. Hillebrand, D. M. Bennett, M. W. Cadotte, Consequences of dominance: A review of evenness effects on local and regional ecosystem processes. *Ecology* **89**, 1510–1520 (2008).

33. D. F. Doak, D. Bigger, E. K. Harding, M. A. Marvier, R. E. O'Malley, D. Thomson, The statistical inevitability of stability-diversity relationships in community ecology. *Am. Nat.* **151**, 264–276 (1998).

34. F. T. Maestre, A. P. Castillo-Monroy, M. A. Bowker, R. Ochoa-Hueso, Species richness effects on ecosystem multifunctionality depend on evenness, composition and spatial pattern. *J. Ecol.* **100**, 317–330 (2011).

35. Y. Zhang, H. Y. H. Chen, P. B. Reich, Forest productivity increases with evenness, species richness and trait variation: A global meta-analysis. *J. Ecol.* **100**, 742–749 (2012).

36. I. Hordijk, D. S. Maynard, S. P. Hart, M. Lidong, H. ter Steege, J. Liang, S. de-Miguel, G. J. Nabuurs, P. B. Reich, M. Abegg, C. Y. A. Yao, G. Alberti, A. M. A. Zambrano, B. V. Alvarado, A. D. Esteban, P. Alvarez-Loayza, L. F. Alves, C. Ammer, C. Antón-Fernández, A. Araujo-Murakami, L. Arroyo, V. Avitabile, G. A. A. Corredor, T. Baker, R. Bałazy, O. Banki, J. Barroso, M. L. Bastian, J. F. Bastin, L. Birigazzi, P. Birnbaum, R. Bitariho, P. Boeckx, F. Bongers, O. Bouriaud, P. H. S. Brancalion, S. Brandl, R. Brienen, E. N. Broadbent, H. Bruelheide, F. Bussotti, R. C. Gatti, R. G. César, G. Cesljar, R. Chazdon, H. Y. H. Chen, C. Chisholm, E. Cienciala, C. J. Clark, D. B. Clark, G. Colletta, D. Coomes, F. C. Valverde, J. J. Corral-Rivas, P. Crim, J. Cumming, S. Dayanandan, A. L. de Gasper, M. Decuyper, G. Derroire, B. DeVries, I. Djordjevic, A. Iêda, A. Dourdain, E. O. N. Laurier, B. Enquist, T. Eyre, A. B. Fandohan, T. M. Fayle, L. V. Ferreira, T. R. Feldpausch, L. Finér, M. Fischer, C. Fletcher, L. Frizzera, J. G. P. Gamarra, D. Gianelle, H. B. Glick, D. Harris, A. Hector, A. Hemp, G. Hengeveld, B. Hérault, J. Herbohn, A. Hillers, E. N. H. Coronado, C. Hui, H. Cho, T. Ibanez, I. B. Jung, N. Imai, A. M. Jagodzinski, B. Jaroszewicz, V. Johanssen, C. A. Joly, T. Jucker, V. Karminov, K. Kartawinata, E. Kearsley, D. Kenfack, D. Kennard, S. Kepfer-Rojas, G. Keppel, M. L. Khan, T. Killeen, H. S. Kim, K. Kitayama, M. Köhl, H. Korjus, F. Kraxner, D. Laarmann, M. Lang, S. Lewis, H. Lu, N. Lukina, B. Maitner, Y. Malhi, E. Marcon, B. S.

Marimon, B. H. Marimon-Junior, A. R. Marshall, E. Martin, O. Martynenko, J. A. Meave, O. Melo-Cruz, C. Mendoza, C. Merow, S. Miscicki, A. M. Mendoza, V. Moreno, S. A. Mukul, P. Mundhenk, M. G. Nava-Miranda, D. Neill, V. Neldner, R. Nevenic, M. Ngugi, P. A. Niklaus, J. Oleksyn, P. Ontikov, E. Ortiz-Malavasi, Y. Pan, A. Paquette, A. Parada-Gutierrez, E. Parfenova, M. Park, M. Parren, N. Parthasarathy, P. L. Peri, S. Pfautsch, O. L. Phillips, N. Picard, M. T. Piedade, D. Piotto, N. C. A. Pitman, I. Polo, L. Poorter, A. D. Poulsen, J. R. Poulsen, H. Pretzsch, F. R. Arevalo, Z. Restrepo-Correa, M. Rodeghiero, S. Rolim, A. Roopsind, F. Rovero, E. Rutishauser, P. Saikia, C. Salas-Eljatib, P. Schall, D. Schepaschenko, M. Scherer-Lorenzen, B. Schmid, J. Schöngart, E. B. Searle, V. Šebeň, J. M. Serra-Diaz, D. Sheil, A. Shvidenko, J. Silva-Espejo, M. Silveira, J. Singh, P. Sist, F. Slik, B. Sonké, A. F. Souza, K. Stereńczak, J. C. Svenning, M. Svoboda, B. Swanepoel, N. Targhetta, N. Tchebakova, R. Thomas, E. Tikhonova, P. Umunay, V. Usoltsev, R. Valencia, F. Valladares, F. van der Plas, D. V. Tran, M. E. Van Nuland, R. V. Martinez, H. Verbeeck, H. Viana, A. C. Vibrans, S. Vieira, K. von Gadow, H. F. Wang, J. Watson, G. D. A. Werner, S. K. Wiser, F. Wittmann, V. Wortel, R. Zagt, T. Zawila-Niedzwiecki, C. Zhang, X. Zhao, M. Zhou, Z. X. Zhu, I. C. Zo-Bi, T. W. Crowther, Evenness mediates the global relationship between forest productivity and richness. *J. Ecol.* **111**, 1308–1326 (2023).

37. X. Lu, J. Mo, F. S. Gilliam, G. Zhou, Y. Fang, Effects of experimental nitrogen additions on plant diversity in an old-growth tropical forest. *Glob. Change Biol.* **16**, 2688–2700 (2010).

38. N. DeMalach, Toward a mechanistic understanding of the effects of nitrogen and phosphorus additions on grassland diversity. *Perspect. Plant Ecol. Evol. Syst.* **32**, 65–72 (2018).

39. S. J. H. Wright, Kyle, "Insights from a long-term, factorial nitrogen, phosphorus, and potassium addition experiment conducted in a mature lowland tropical forest in the barro colorado nature monument" in *The First 100 Years of Research on Barro Colorado: Plant and Ecosystem Science,* H. W. Muller-Landau, S. Joseph, Ed. (Smithsonian Institution Scholarly Press, 2024), vol. 2, pp. 000–000.

40. P. M. Vitousek, Litterfall, nutrient cycling, and nutrient limitation in tropical forests. *Ecology* **65**, 285–298 (1984).

41. P. A. Sanchez, *Properties and Management of Soils in the Tropics* (Wiley, 1976).

42. T. W. Walker, J. K. Syers, The fate of phosphorus during pedogenesis. *Geoderma* **15**, 1–19 (1976).

43. A. F. Harrison, *Soil Organic Phosphorus: A Review of World Literature* (CAB International, 1987).

44. X. Liu, D. Burslem, J. D. Taylor, A. F. S. Taylor, E. Khoo, N. Majalap-Lee, T. Helgason, D. Johnson, Partitioning of soil phosphorus among arbuscular and ectomycorrhizal trees in tropical and subtropical forests. *Ecol. Lett.* **21**, 713–723 (2018).

45. G. K. Phoenix, D. A. Johnson, S. P. Muddimer, J. R. Leake, D. D. Cameron, Niche differentiation and plasticity in soil phosphorus acquisition among co-occurring plants. *Nat. Plants* **6**, 349–354 (2020).

46. Y. Chen, M. Liang, D. F. R. P. Burslem, D. Johnson, S. Yu, X. Liu, Contrasting response of root traits of arbuscular mycorrhizal and ectomycorrhizal trees to phosphorus availability in subtropical forests. *Plant Soil* **507**, 519–531 (2025).

47. M. Liang, X. Zhang, J. Zhang, X. Liu, Different phosphorus preferences among arbuscular and ectomycorrhizal trees in a subtropical forest. *Soil Biol. Biochem.* **194**, 109448 (2024).

48. Y. Li, D. Tian, H. Yang, S. Niu, Y. Luo, Size-dependent nutrient limitation of tree growth from subtropical to cold temperate forests. *Funct. Ecol.* **32**, 95–105 (2018).

49. S. Alvarez-Clare, M. C. Mack, M. Brooks, A direct test of nitrogen and phosphorus limitation to net primary productivity in a lowland tropical wet forest. *Ecology* **94**, 1540–1551 (2013).

50. M. N. Umana, J. Needham, C. Fortunel, From seedlings to adults: Linking survival and leaf functional traits over ontogeny. *Ecology* **106**, e4469 (2025).

51. P. Chesson, Mechanisms of maintenance of species diversity. *Annu. Rev. Ecol. Syst.* **31**, 343–366 (2000).

52. F. Huang, M. Liang, Y. Zheng, X. Liu, Y. Chen, W. Li, S. Luo, S. Yu, Soil nitrogen availability intensifies negative density-dependent effects in a subtropical forest. *J. Plant Ecol.* **13**, 281–287 (2020).

53. J. S. Wright, Plant diversity in tropical forests: A review of mechanisms of species coexistence. *Oecologia* **130**, 1–14 (2002).

54. H. Hishe, L. Oosterlynck, K. Giday, W. De Keersmaecker, B. Somers, B. J. F. E. Muys, A combination of climate, tree diversity and local human disturbance determine the stability of dry Afromontane forests. *For. Ecosyst.* **8**, 16 (2021).

55. E. Laliberté, P. Legendre, A distance-based framework for measuring functional diversity from multiple traits. *Ecology* **91**, 299–305 (2010).

56. D. Craven, N. Eisenhauer, W. D. Pearse, Y. Hautier, F. Isbell, C. Roscher, M. Bahn, C. Beierkuhnlein, G. Bonisch, N. Buchmann, C. Byun, J. A. Catford, B. E. L. Cerabolini, J. H. C. Cornelissen, J. M. Craine, E. De Luca, A. Ebeling, J. N. Griffin, A. Hector, J. Hines, A. Jentsch, J. Kattge, J. Kreyling, V. Lanta, N. Lemoine, S. T. Meyer, V. Minden, V. Onipchenko, H. W. Polley, P. B. Reich, J. van Ruijven, B. Schamp, M. D. Smith, N. A. Soudzilovskaia, D. Tilman, A. Weigelt, B. Wilsey, P. Manning, Multiple facets of biodiversity drive the diversity-stability relationship. *Nat. Ecol. Evol.* **2**, 1579–1587 (2018).

57. L. Jiang, S. Wan, L. Li, Species diversity and productivity: Why do results of diversity-manipulation experiments differ from natural patterns? *J. Ecol.* **97**, 603–608 (2009).

58. B. J. McGill, R. S. Etienne, J. S. Gray, D. Alonso, M. J. Anderson, H. K. Benecha, M. Dornelas, B. J. Enquist, J. L. Green, F. He, A. H. Hurlbert, A. E. Magurran, P. A. Marquet, B. A. Maurer, A. Ostling, C. U. Soykan, K. I. Ugland, E. P. White, Species abundance distributions: Moving beyond single prediction theories to integration within an ecological framework. *Ecol. Lett.* **10**, 995–1015 (2007).

59. J. P. Grime, Benefits of plant diversity to ecosystems: Immediate, filter and founder effects. *J. Ecol.* **86**, 902–910 (1998).

60. M. J. Wassen, H. O. Venterink, E. D. Lapshina, F. Tanneberger, Endangered plants persist under phosphorus limitation. *Nature* **437**, 547–550 (2005).

61. T. Ceulemans, C. J. Stevens, L. Duchateau, H. Jacquemyn, D. J. Gowing, R. Merckx, H. Wallace, N. van Rooijen, T. Goethem, R. Bobbink, E. Dorland, C. Gaudnik, D. Alard, E. Corcket, S. Muller, N. B. Dise, C. Dupre, M. Diekmann, O. Honnay, Soil phosphorus constrains biodiversity across European grasslands. *Glob. Change Biol.* **20**, 3814–3822 (2014).

62. Z. Fang, H. Yu, C. Li, B. Wang, F. Jiao, J. Huang, Long-term phosphorus addition alters plant community composition but not ecosystem stability of a nitrogen-enriched desert steppe. *Sci. Total Environ.* **879**, 163033 (2023).

63. N. Zong, G. Hou, P. Shi, T. Zhou, J. Yu, J. Tian, Different responses of community temporal stability to nitrogen and phosphorus addition in a non-degraded alpine grassland. *Ecol. Indic.* **143**, 109310 (2022).

64. P. M. Vitousek, S. Porder, B. Z. Houlton, O. A. Chadwick, Terrestrial phosphorus limitation: Mechanisms, implications, and nitrogen–phosphorus interactions. *Ecol. Appl.* **20**, 5–15 (2010).

65. X. Gan, W. Li, X. Ye, Y. Jiang, C. Zhao, Nutrient enrichment weakens community temporal stability via asynchrony and species dominance in a subalpine grassland. *Agric. Ecosyst. Environ.* **379**, 109358 (2025).

66. B. B. Lamont, P. K. Groom, Seeds as a source of carbon, nitrogen, and phosphorus for seedling establishment in temperate regions: A synthesis. *Am. J. Plant Sci.* **4**, 30–40 (2013).

67. M. R. Leishman, M. Westoby, The role of seed size in seedling establishment in dry soil conditions—Experimental evidence from semi-arid species. *J. Ecol.* **82**, 249–258 (1994).

68. R. P. Phillips, E. Brzostek, M. G. J. N. P. Midgley, The mycorrhizal-associated nutrient economy: A new framework for predicting carbon–nutrient couplings in temperate forests. *New Phytol.* **199**, 41–51 (2013).

69. L. Tedersoo, M. Bahram, M. Zobel, How mycorrhizal associations drive plant population and community biology. *Science* **367**, eaba1223 (2020).

70. J. A. Bennett, H. Maherali, K. O. Reinhart, Y. Lekberg, M. M. Hart, J. Klironomos, Plant-soil feedbacks and mycorrhizal type influence temperate forest population dynamics. *Science* **355**, 181–184 (2017).

71. M. Liang, D. Johnson, D. F. R. P. Burslem, S. Yu, M. Fang, J. D. Taylor, A. F. S. Taylor, T. Helgason, X. Liu, Soil fungal networks maintain local dominance of ectomycorrhizal trees. *Nat. Commun.* **11**, 2636 (2020).

72. R. J. Payne, N. B. Dise, C. D. Field, A. J. Dore, S. J. M. Caporn, C. J. Stevens, Nitrogen deposition and plant biodiversity: Past, present, and future. *Front. Ecol. Environ.* **15**, 431–436 (2017).

73. Y. Ke, Q. Yu, H. Wang, Y. Zhao, X. Jia, Y. Yang, Y. Zhang, W. Zhou, H. Wu, C. Xu, T. Sun, Y. Gao, A. Jentsch, N. He, G. Yu, The potential bias of nitrogen deposition effects on primary productivity and biodiversity. *Glob. Change Biol.* **29**, 1054–1061 (2023).

74. X. Liu, M. Liang, R. S. Etienne, Y. Wang, C. Staehelin, S. Yu, Experimental evidence for a phylogenetic Janzen–Connell effect in a subtropical forest. *Ecol. Lett.* **15**, 111–118 (2012).

75. J. J. Gonzalez Medeiros, B. Perez Cid, E. Fernandez Gomez, Analytical phosphorus fractionation in sewage sludge and sediment samples. *Anal. Bioanal. Chem.* **381**, 873–878 (2005).

76. E. Pebesma, Multivariable geostatistics in S: The gstat package. *Comput. Geosci.* **30**, 683–691 (2004).

77. H. Tuomisto, An updated consumer's guide to evenness and related indices. *Oikos* **121**, 1203–1218 (2012).

78. M. Loreau, C. de Mazancourt, Species synchrony and its drivers: Neutral and nonneutral community dynamics in fluctuating environments. *Am. Nat.* **172**, E48–E66 (2008).

79. F. Cribari-Neto, A. Zeileis, Beta regression in R. *J. Stat. Softw.* **34**, 1–24 (2010).

80. J. S. Lefcheck, R. Freckleton, piecewiseSEM: Piecewise structural equation modelling in r for ecology, evolution, and systematics. *Methods Ecol. Evol.* **7**, 573–579 (2016).

81. B. Shipley, Confirmatory path analysis in a generalized multilevel context. *Ecology* **90**, 363–368 (2009).

82. R. Bivand, G. Millo, G. Piras, A review of software for spatial econometrics in R. *Mathematics* **9**, 10.3390/math9111276 (2021).

83. D. Bates, M. Maechler, B. Bolker, S. Walker, R. H. Bojesen, H. Singmann, B. Dai, F. Scheipl, G. Grothendieck, G. Grothendieck, P. Green, J. Fox, A. Bauer, P. N. Krivitsky, E. Tanaka, M. Jagan, R. D. Boylan, A. Ly, Package 'lme4'. *Convergence* **12**, 2 (2015).

84. A. Baselga, C. D. L. Orme, betapart: An R package for the study of beta diversity. *Methods Ecol. Evol.* **3**, 808–812 (2012).

85. R Core Team, R: A Language and Environment for Statistical Computing (R Foundation for Statistical Computing, Vienna, Austria, 2022); https://www.R-project.org/.

86. J. A. LaManna, S. A. Mangan, A. Alonso, N. A. Bourg, W. Y. Brockelman, S. Bunyavejchewin, L. W. Chang, J. M. Chiang, G. B. Chuyong, K. Clay, R. Condit, S. Cordell, S. J. Davies, T. J. Furniss, C. P. Giardina, I. Gunatilleke, C. V. S. Gunatilleke, F. He, R. W. Howe, S. P. Hubbell, C. F. Hsieh, F. M. Inman-Narahari, D. Janik, D. J. Johnson, D. Kenfack, L. Korte, K. Kral, A. J. Larson, J. A. Lutz, S. M. McMahon, W. J. McShea, H. R. Memiaghe, A. Nathalang, V. Novotny, P. S. Ong, D. A. Orwig, R. Ostertag, G. G. Parker, R. P. Phillips, L. Sack, I. F. Sun, J. S. Tello, D. W. Thomas, B. L. Turner, D. M. Vela Diaz, T. Vrska, G. D. Weiblen, A. Wolf, S. Yap, J. A. Myers, Plant diversity increases with the strength of negative density dependence at the global scale. *Science* **356**, 1389–1392 (2017).

87. F. Hegyi, A simulation model for managing jack-pine stands simulation. *RoyalColl. For. Res. Ther. Notes* **30**, 74–90 (1974).

88. G. Zhou, G. Yin, X. Tang, D. Wen, C. Liu, Y. Kuang, W. Wang, *Forest Ecosystem Carbon Stocks in China—Biomass Equations* (Beijing Science Press, 2018).
